# Physiology and Physical Chemistry of Bile Acids

**DOI:** 10.3390/ijms22041780

**Published:** 2021-02-10

**Authors:** Maria Chiara di Gregorio, Jacopo Cautela, Luciano Galantini

**Affiliations:** 1 Department of Molecular Chemistry and Materials Science, Weizmann Institute of Science, Rehovot 7610001, Israel; 2 Department of Chemistry, Sapienza University of Rome, 00185 Rome, Italy; jacopo.cautela@uniroma1.it

**Keywords:** bile acids, physiological functions, bile acid derivatives, pharmacological application, material science applications, self-assembly, surfactants

## Abstract

Bile acids (BAs) are facial amphiphiles synthesized in the body of all vertebrates. They undergo the enterohepatic circulation: they are produced in the liver, stored in the gallbladder, released in the intestine, taken into the bloodstream and lastly re-absorbed in the liver. During this pathway, BAs are modified in their molecular structure by the action of enzymes and bacteria. Such transformations allow them to acquire the chemical–physical properties needed for fulling several activities including metabolic regulation, antimicrobial functions and solubilization of lipids in digestion. The versatility of BAs in the physiological functions has inspired their use in many bio-applications, making them important tools for active molecule delivery, metabolic disease treatments and emulsification processes in food and drug industries. Moreover, moving over the borders of the biological field, BAs have been largely investigated as building blocks for the construction of supramolecular aggregates having peculiar structural, mechanical, chemical and optical properties. The review starts with a biological analysis of the BAs functions before progressively switching to a general overview of BAs in pharmacology and medicine applications. Lastly the focus moves to the BAs use in material science.

## 1. Introduction

Bile acids (BAs) constitute an important class of biological molecules produced in the metabolism of all vertebrates. In mammals, they exhibit the so called C24 structure: 24 carbon atoms form a steroid nucleus (three six-member rings indicated as A, B C and a five-member ring indicated as D) and a five-carbon side chain with a carboxyl group at the C-24 position.

**Figure 1:**
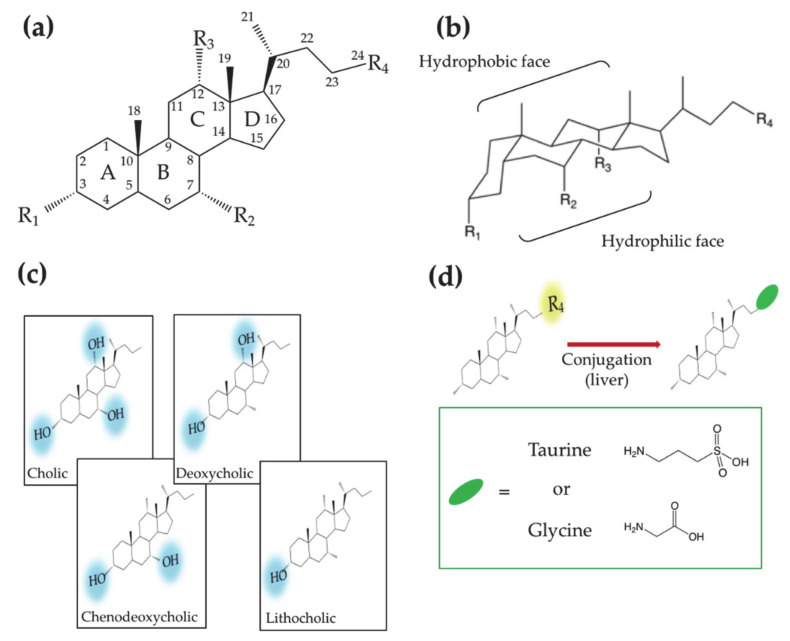
(**a**) Planar representation of the general molecular structure of bile acids (Bas). Letters, numbers and labels R_i_ indicate the rings of the steroid skeleton, the carbon atoms and the functional groups, respectively. (**b**) Chair representation of the general molecular structure of BAs. Brackets indicate the hydrophobic and hydrophilic faces. (**c**) Molecular structures of BAs showing different hydroxyl groups on the steroid backbone. (**d**) Molecular structures of the aminoacid conjugated BAs.

The A and B rings are linked in *cis* configuration, inducing an overall bent shape. Such a structural feature delineates a concave and convex side of the steroidal backbone where OH groups in α orientation (up to three) and two methyl groups in β orientation, respectively, point out. Therefore, two opposite faces with hydrophilic and hydrophobic properties can be distinguished. (Figure 1a,b). Further variations of the molecular structure can be observed at C-3 carbon due to hydroxyl, sulfate or glucuronate substituents [1,2]. C-6 and C-24 glucoronide conjugates were also found in humans [2]. Other C-24 substituents are glycine or taurine [3,4,5,6]. Recently Dorrestein et al. reported new amino acid C-24 substituted cholic acid (CA) namely phenylalanocholic, tyrosocholic and leucocholic acid [7]. BA actions generally occur in conditions where they are deprotonated; for this reason, many authors refer to them as bile salts instead of acids. In this review the term BA will be used keeping in mind that we refer mostly to their salt form.

According to the order in which they are produced in the human body, BAs are differently named. CA and chenodeoxycholic (CDCA) acids—3 and 2 OH groups, respectively—are first synthesized by the hepatocyte and thus named primary BAs. Subsequently CA and CDCA are conjugated to glycine or taurine, giving rise to glycocholic (GCA), taurocholic (TCA) acids and glycochenodeoxycholic (GCDCA), taurochenodeoxycholic (TCDCA) acids, respectively. Further metabolization leads to the secondary BAs, deoxycholic (DCA) and lithocholic (LCA) acids, that present two and one OH groups, respectively (Figure 1c,d). The increase in the hydrophobic character, moving from primary to secondary BAs, affects the BA chemical–physical and physiological properties, making them differently active in the diverse parts of the enterohepatic circuit.

Generally speaking, five major physiological roles can be distinguished in BA activity: (1) regulation of cholesterol homeostasis; (2) deterrence for the formation of gallstones and kidney stones; (3) emulsification of dietary lipids and absorption of fat-soluble vitamins; (4) antimicrobial activity; (5) regulation function.

These functions will be described in the next paragraphs, following the BA physiological pathway—from biosynthesis to elimination/recirculation.

BAs are produced in the liver and stored in the gallbladder. Subsequently they are secreted through the biliary tract in the intestine, absorbed in the intestinal epithelium to pass into the portal circulation and return to the liver. The overall process is named enterohepatic circulation [8]. A small part of BAs escapes this cycle being secreted through the feces. The daily loss of BAs is compensated by new liver production.

## 2. Synthesis in the Liver and Storage in the Gallbladder: BAs in Cholesterol Metabolism

BAs are produced in their primary forms (CA and CDCA) in the liver, adopting cholesterol as starting substrate and the activity of 16 enzymes. Such enzymes catalyze 17 different reactions. In humans, the synthesis takes place in multiple intracellular compartments such as the cytosol, endoplasmic reticulum, mitochondria and peroxisomes.

A living organism can exploit two different synthetic routes for the BA synthesis [9].

The route, that is quantitively more important for adult humans, is known as “classic or neutral path” and provides for more than 90% of the BA needs. Such a synthetic pathway starts with the hydroxylation of the sterol ring on C7 mediated by the cholesterol 7α-hydroxylase (CYP7A1). Subsequently the intermediate is immediately modified on the lateral chain by the sterol 27-hydroxylase (CYP27) for the CDCA synthesis or, in the case of the CA synthesis, is further hydroxylated on C-12 by the 12α-hydroxylase (CY8B1) before the CYP27 catalyzed step. In this pathway, the CYP7A1 activity is the kinetic key that determines the overall rate of the metabolic pathway. On the other hand, the CY8B1 activity, modulating the hydrophilicity composition of the steroid nucleus, controls the CA and CDCA production ratio.

The second biosynthetic route, named “acidic path”, leads to the production of CDCA in humans and it is dominant in human neonates. In this pathway the chemical modifications of the cholesterol substrate involve first the lateral chain where a hydroxyl group is introduced on C27 by the CYP27 activity. Subsequently the modification of the sterol part is performed by the oxysterol 7α-hydroxylase (CYP7B1). A minority fraction of C25- and C24-hydroxycholesterols generated by the corresponding hydroxylases can also enter the acidic pathway to form BAs. Although most of the hydroxycholesterols are produced in the liver, hydroxycholesterols generated in extra hepatic tissues, such as lungs and brain, may be transported to the liver and be also involved in this BAs biosynthetic route. After their synthesis, CA and CDCA are conjugated to taurine and glycine by the activity of two enzymes, bile acid-CoA synthase and bile acid-CoAamino acid N-acyltransferase. The conjugation makes the produced BA more hydrophilic and more acidic on the side chain: the pKa decreases from ~5.0 to ~3.9 and <2 for glycine and taurine conjugates, respectively. The amount of synthesized BA is regulated by control mechanisms that operate at transcriptional level, where the transcription factors are nuclear receptors. An exceeding amount of BA triggers a negative feedback mechanism, starting with the BAs binding to the hepatic farnesoid X receptor (FXR) and ending with the inhibition of the genes expressing the CYP7A1 and CYP8B1 enzymes involved in the biosynthesis of BA [10,11]. The activation of FXR affects not only the synthesis itself but it also regulates the level of the BA in the intestine and biliary tree. Indeed, in order to assure concurrently an efficient lipid absorption and a sustainable hepatic level of BA, the FXR increases the expression of the transporters which mediate the efflux of the BA into the biliary canaliculi. At the same time, it suppresses the expression of the importer NTCP, thus reducing the BA reabsorption from the blood into the hepatocytes [12]. Besides the effect in the liver, reabsorbed BAs can bind also the intestinal FXR in the distal ileum, activating it. The activated FXR stimulates in turn the expression of the fibroblast growth factor 19 (FGF19) and its release into the portal blood. Once it reaches the hepatocyte plasma membrane, FGF19 binds the FGF receptor 4 (FGFR4)/β-klotho complex, triggering a signaling cascade that results in the suppression of the CYP7A1 mRNA and thus the suppression of the CYP7A1 expression [13,14].

BA production utilizes a consistent amount of the cholesterol pool (about 500 mg per day), thus turning out to be one of the main mechanisms for cholesterol regulation in the human body. [15] Consistently, hypercholesterolemia is often treated by Bas sequestrants [16,17,18,19]. It has been observed that certain animals when fed with hypercholesterolic diet manage to keep normal the plasma cholesterol level, thanks to a compensating mechanism that increases the production of BAs [20]. BAs sequestrants [16] are generally formed by cationic polyelectrolytes, which are able to bind the BAs through both electrostatic and hydrophobic attractive interactions and as such to remove them from the enterohepatic circulation. Sequestration stimulation increased conversion of cholesterol into BAs in the liver, thereby leading to the lowering of the LDL (low-density lipoproteins, “bad”) cholesterol in the blood, and simultaneously to an increase in the HDL (high-density lipoproteins, “good”) cholesterol and triglycerides [21,22]. BA sequestrants are also used in the medical treatment of BA related diseases, such as BA diarrhea caused by BAs in the large intestine or colon due to the malabsorption of BA in the small intestine (secondary BA diarrhea) or BA overproduction resulting from defective feedback inhibition of the biosynthesis of BAs in the liver (primary BA diarrhea) [23,24,25,26]. In the treatment, sequestrants bind BAs, hinder them from contact with the colonic mucosa, thereby decreasing the BA level in the colon [27,28,29]. BA sequestrants can also reduce glucose levels in patients with type 2 diabetes mellitus, although the mechanism of action still remains unclear [30]. Moreover, BAs malabsorption reduces the amount of BA in the small intestine inducing fat maldigestion and consequent steatorrhea as occurring in patients with short bowel syndrome [31]. Consequently, the maldigested fatty acids in the small intestine complex the luminal Ca, lowering the fraction of Ca available for the dietary oxalate precipitation. In turn, the oxalate remains as free ions and is hyperabsorbed by the colon, leading to hyperoxaluria and increasing the possibility of kidney stone formation. Oral therapies based on natural and synthetic conjugated bile salts were observed to decrease fecal fat and urinary oxalate execration in patients with short bowel syndrome [32,33].

A further important role of BAs towards cholesterol concerns the solubilization activity in the bile. Cholesterol is majorly eliminated through the secretion in bile. Bile is a solution produced in the liver that is composed by 95% of water. Free cholesterol is insoluble in aqueous solutions. However, in bile due to the presence of BAs (about 0.7%) and lipids like lecithin, cholesterol is easily solubilized through the formation of mixed micelles. Such a process avoids the cholesterol supersaturation and in turn the formation of gallstones. The interaction between cholesterol and BAs has been analyzed both from a chemical and medical point of view. In the first case, several studies analyzed the structure, stability and parameter formation of BAs–cholesterol-based micelles and crystals [34,35,36]. In the latter case, CDCA was proved in the 1970s as successful molecule for dissolution of cholesterol gallstones [37,38,39,40]. However, its use was lately abandoned because of the occurrence of side effects. The more hydrophilic ursodeoxycholic acid (UDCA) and its taurine conjugate started to be investigated as alternative treatment for cholelithiasis and their use in oral therapy is still ongoing, albeit restricted to a specific target group of patients (e.g., patients having gallstones due to temporary and non-genetic causes) [41]. The combined efficiency of UDCA and polyunsaturated fatty acids in dissolving cholesterol gallstones in mice was recently reported by Lee et al. [42].

After the synthesis, conjugated BAs are transferred into bile, passing through the hepatocyte’s membrane to the canaliculi via the bile salt export pump. The bile in the canaliculi converge in a series of ducts that eventually terminate into the common hepatic duct. Via the hepatic duct, the bile reaches the gallbladder, where it is stored or is delivered directly to the intestinal lumen [43].

## 3. From the Gallbladder to the Intestine: Lipid Solubilization and Absorption

After a meal, the cholecystokinin hormone is released and its presence is the signal for the gallbladder to release bile. At this point the aminoacidic conjugation is functional for the BA activities since it allows for the transits to the small intestine via the biliary tree. Indeed, being completely ionized at the pH of biliary tract and small intestine, conjugated BAs cannot diffuse through the cell membranes, thus assuring consistent intraluminal concentration in the intestine for lipid digestion. Moreover, the conjugated BAs are less prone than unconjugated BAs to precipitate in presence of high concentration of Ca^2+^ ions. In the distal small intestine, bacteria break the conjugation with the aminoacidic of a fraction of conjugated BAs. Deconjugation is completed in the colon where further modifications as dihydroxylation and epimerization occur to give rise to secondary BAs, i.e., DCA, LCA and UDCA. Recently, anaerobic in vitro reconstitution experiments showed that six enzymes are sufficient for the conversion of cholic acid into DCA [44].

**Figure 2:**
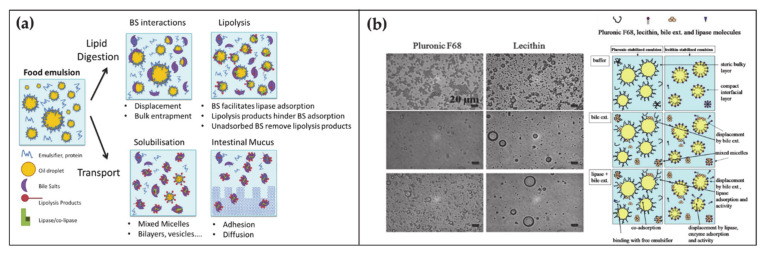
(**a**) Schematic representation of the BA functions and self-assembly during the lipid digestion and transport in the intestine (adapted from Macierzanka, A.; Torcello-Gómez, A.; Jungnickel, C.; Maldonado-Valderrama, J. Bile Salts in Digestion and Transport of Lipids. *Adv. Colloid Interface Sci.* **2019**, 274, 102045. Ref. [46] with permission from (2019) Elsevier). (**b**) Transmission Electron Microscopy images of oil-in-water emulsions stabilized by two surfactants with interest in food and drug industry, namely Pluronic F68 and Lecithin (left top). Transmission Electron Microscopy images of the surfactant-emulsion transformation upon BA (left center) and BA + lipase addition (left bottom). Scheme representing the disposition of surfactant-BA-lipase at the oil/water interface (right). Reproduced from Torcello-Gómez, A.; Maldonado-Valderrama, J.; Martín-Rodríguez, A.; McClements, D.J. Physicochemical Properties and Digestibility of Emulsified Lipids in Simulated Intestinal Fluids: Influence of Interfacial Characteristics. *Soft Matter* **2011**, *7*, 6167–6177. Ref. [53] with permission from The Royal Society of Chemistry.

At any time, a portion of 85–90% of BAs is present in the small intestine. Here BAs have several roles in the digestion of lipids, ranging from emulsification to transport (Figure 2a) [45,46]. Lipid digestion starts in the stomach where the food is initially broken down by the mechanical action of peristalsis and by the chemical activity of gastric juices. Subsequently the digestion continues in the duodenum where the partially digested food mixes with digestive enzymes from the pancreas—pancreatic lipase—and BAs. At this stage, the ingested lipids are in the form of oil-in-water emulsion stabilized by different surface-active substances such as proteins and phospholipids. In order to hydrolyze lipids into simpler and absorbable molecules, lipase and its co-factor co-lipase have to anchor on the droplet surface. The first crucial role of BAs is to increase the bioavailability of the lipid substrates to the enzyme by displacing the different stabilizers at the water–oil interface. Subsequently BAs indirectly help the positioning of lipase on the lipidic substrate by favoring the interfacial adsorption of co-lipase. After the lipase activity, lipids are decomposed in free fatty acids and monoglycerides. These products remain at the oil–water droplet interface until BAs englobe them in mixed micelles, thereby providing their removal and transport through the intestinal mucus.

The fundamental understanding of these mechanisms is supported by a large collection of literature that by means of microscopic, spectroscopic and rheological techniques, (i) analyzed the displacement mechanism of BAs with respect to a large number of proteins and lipids (Figure 2b), (ii) demonstrated the improved activity of lipase in presence of BAs, (iii) elucidated the mechanism of lipid transport through the intestinal mucus [45,47,48,49]. Besides clarifying the physiological mechanism, such knowledges turn out to be essential for food and drug industry in order to engineer efficient strategies for drugs, dietary lipids and sugars uptake in the gastrointestinal tract [50,51,52,53].

## 4. Antimicrobial Activity

**Figure 3:**
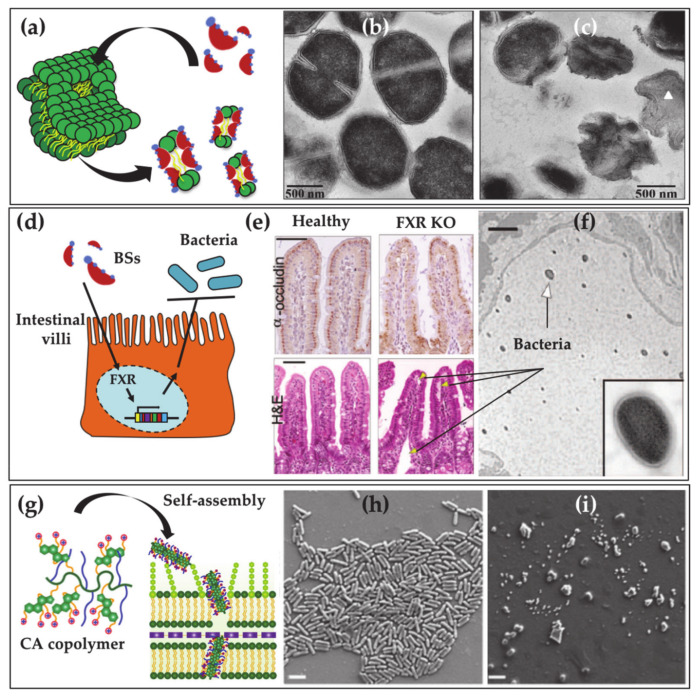
(**a**) Direct antimicrobial mechanism of natural BA: detergent effect of BA on the bacterial membrane. Transmission Electron Microscopy images of *S. aureus* before (**b**) and after (**c**) the interaction with glycocholic acid (GCA) (Sannasiddappa, T.H.; Lund P.A.; Clarke S.R. In Vitro Antibacterial Activity of Unconjugated and Conjugated Bile Salts on *Staphylococcus aureus*. *Front. Microbiol.* **2017**, *8*, 1581 [99] copyright © 2017 Sannasiddappa, Lund, Clarke (CCBY)) (**d**) Indirect antimicrobial mechanism of natural BA: BAs activate the farnesoid X receptor (FXR) that in turn induces the expression of genes producing toxic molecules for bacteria (Inagaki, T.; Moschetta, A.; Lee, Y.K.; Peng, L.; Zhao, G.; Downes, M.; Yu, R.T.; Shelton, J.M.; Richardson, J.A.; Repa, J.J.; et al. Regulation of Antibacterial Defense in the Small Intestine by the Nuclear Bile Acid Receptor. *Proc. Natl. Acad. Sci. USA* **2006**, *103*, 3920–3925. Ref. [64], Copyright (2006) National Academy of Sciences, U.S.A.). (**e**) Transverse sections of terminal ileum of mice immunostained with anti-occludin antisera (top) and hematoxylin and eosin (H&E, bottom), scale bar 50 µm. Micrographs of control mice (left) are contraposed to micrographs of FXR knockout mice (right). (**f**) Lymphatic vessel section of FXR knockout mice, scale bar 2 µm. Arrows point to traces of edema and dilated lymphatic vessels that are induced by bacteria. (**g**) Copolymers of cholic acid (CA) and polyethylene glycol self-assemble into rods that are able to penetrate the bacterial membrane Scanning Electron Microscopy images of *E.coli* without (**h**) and with (**i**) BA polymer treatment (adapted with permission from Rahman, M.A.; Jui, M.S.; Bam, M.; Cha, Y.; Luat, E.; Alabresm, A.; Nagarkatti, M.; Decho, A.W.; Tang, C. Facial Amphiphilicity-Induced Polymer Nanostructures for Antimicrobial Applications *ACS Appl. Mater. Interfaces* **2020**, *12*, 21221–21230 [105]. Copyright (2020) American Chemical Society).

BAs and gut microbiota have a mutual interaction. As aforementioned, the molecular structures of primary BAs are modified by the gut bacteria to give rise to secondary BAs or other BA forms that escape the enterohepatic circulation. Nevertheless, also BAs influence the microbiota composition [54,55]. Concerning the latter point, it has been for example observed that low levels of BAs in the gut induce an overgrowth of bacteria and potential pathogens, increasing the occurrence of inflammations and bacterial translocation. This is due to the fact that BAs have relevant antimicrobial activities. The BAs antimicrobial activity can be mainly related to two different action mechanisms. The first one refers to the detergent properties of BAs and their ability to penetrate and break membranes (Figure 3a–c). In vitro experiments proved that BAs can provoke hemolysis in erythrocytes due to BA-induced membrane damages [56]. Similarly, cells were observed to shrivel under BAs exposure and release intracellular materials [57,58,59]. Fast and drastic decomposition of membrane proteins occurs at high BA concentrations [60]. Sub micellar BA concentrations can also alter membrane permeability by interacting with membrane-bounded enzymes/proteins, by changing the transmembrane flux of divalent cations or by inducing hydrophobicity and external charge modification of the cells [61]. Antimicrobial activity due to detergent properties is particularly strong in unconjugated BAs [62]. Indeed, unconjugated BAs can passively penetrate through the lipid bilayer by the “flip-flop” mechanism and access the cells [63]. The kinetic of the penetration process is strongly affected by the number of hydroxyl groups, becoming more efficient as the number of hydroxyl group decreases. Consistently antimicrobial activity of DCA was proved in vitro to be an order of magnitude higher than that of CA. On the other hand, conjugated BAs are strong acids and are fully ionized at physiological pHs. In the absence of a specific transport system, this feature inhibits the BA penetration through the membrane, favoring instead the BA adhesion on the external part of the bilayers. In vitro studies showed that such adhesion varies the membrane surface properties that, although destabilizing cell integrity, induce less membrane damage than the unconjugated analogues. Despite the in vitro results, in vivo experiments suggested relevant antibacterial properties related to the presence of conjugated BAs. It was for example observed that events that decrease conjugated BA secretion in animals, such as liver cirrhosis or bile duct ligation, induce an increase in the bacterial growth (Figure 3d–f). Similarly, upon feeding of conjugated BAs and bile in BAs-deficient intestines, overgrowth of bacteria was suppressed. Such contradiction was solved by Inagaki and coworkers in 2006 who showed an alternative antibacterial mechanism of BAs [64]. It was proved that conjugated BAs are natural ligands of the nuclear receptor farnesoid X (FXR) that in turn activate the expression of genes whose products (e.g., nitric oxide) stop bacterial overgrowth. Such a mechanism occurs in the distal small intestine that turns out to host a poor fraction of microbes (about 10^4^ to 10^5^ colony-forming units/mL), a high concentration of conjugated BAs (about 10 mM during digestion time) and an FXR level three times higher than the epithelium of the proximal small intestine.

Moreover Kang et al. recently discovered that the bile acid 7a-dehydroxylating gut bacteria, responsible for the biotransformation of primary BAs into secondary BAs, secrete tryptophan and proline-based antibiotics. Such antibiotics are able to obstruct bacterial pathogens causing diarrhea and colitis and their efficiency is enhanced in presence of DCA and LCA [65]. Commensal gut bacteria were also demonstrated to have a regulating function towards liver cancer by using BAs as signaling molecules. Indeed, BAs through the portal vein reach the liver sinusoidal endothelial cells and regulate the accumulation of natural killer T cells, which in turn inhibits the liver tumor growth. It was found in particular that the accumulation of the natural killer T cells was favored by primary BAs and disfavored by secondary ones [66].

To conclude this paragraph, it has to be emphasized that the enteric flora in the human body can have protecting functions, by for example eliciting immune responses, but can be also responsible for pathologies such as inflammatory bowel disease and cancers [67,68]. Therefore, the resistance of bacteria to the bile toxicity turns out to be one of the key mechanisms used by the human body to select probiotic strains that can positively perform in the gastrointestinal tract. The functions showed in the physiological environment can be modified/amplified by covalently or not-covalently combining the natural BAs with other moieties/molecules. The huge possibilities offered by modern organic chemistry has made the BAs functionalization more and more complex. Novel synthetic procedures have allowed for precise control on stereospecific substitutions, polymerization processes, introduction of differently charged groups and derivatization of concurrent and multiple positions on the original molecular structure.

The BA ability to penetrate membranes has been boosted up in derivatives, enabling for preparation of active molecule carriers where the BA derivatives (BADs) can work both as monomer (e.g., by the specific interaction with proteins membrane) or in aggregates (e.g., by the formation of drugs including transferosomes, vesicles, micelles, gels). BAs emulsification properties have been investigated to formulate new drugs for treatment of obesity and other diseases related to slow fat assimilation and high levels of cholesterol (e.g., lithiasis). Starting from the regulatory functions of BAs, BADs have also been developed to interfere in several physiological pathways (e.g., glucose, lipid and energy metabolism) for the treatment of metabolic syndromes.

## 5. Functionalized BAs in Medicine

In the light of their biological origin and the specific interaction with several physiological pathways, BA and BADs have naturally become the protagonist of many biomedical and pharmaceutical studies [69]. For example, antimicrobial activity of natural BAs has been used in traditional Chinese Medicine in environments different to the physiological one, e.g., in treatment of skin laceration or for reduction in swelling, pain and fever [70].

Both natural BAs and a large number of BADs have been proved efficient against many bacteria, parasites, fungi and to induce apoptosis in different types of cancerogenic cells. To mention some examples, it has been known for more than a decade that LCA induces apoptosis in neuroblastoma [71], breast cancer [72], prostate cancer [73] cells, although recently its selective effects on nephroblastoma and sarcoma cell-lines was questioned [74]. UDCA turned out to both favor and block apoptotic processes in different types of cells, according to the dosage and administration time. At low dose, UDCA efficiency has been proved in blocking lung cancer cell migration and propagation of colon and liver cancer cells. Better performance and lesser occurrence of side-effects have been shown when UDCA is used in combination with other anticancer drugs [75]. BA molecules have also been used as a platform for the synthesis of BA-based anticancer drugs recently overviewed [76]. Synthetic C24 aminoacid conjugated BA such as CDCA and UDCA acids have been reported to induce apoptosis in several human cancer cells like calf pulmonary endothelial cancer cells [77], hepatocellular carcinoma cells [78,79], breast carcinoma cells [80,81], leukemic T cells [82], prostate [83], colon [84] and gastric cancer cells. Piperazinil derivatives of CDCA and UDCA has been proved to be active anticancer drugs [85]. Heparin containing C24 modified DCA was reported to be able to limit migration and adhesion of cancer cells to extracellular matrix and to inhibit formation of metastasis [86,87]. Functionalization at C3, C7 and C12 with groups containing positive trimethyl ammonium heads was observed to show enhanced cytotoxicity compared to the precursor BA [88]. C24 substituted CA derivatives containing phenyl, benzothiazole, and four methylphenyl groups via aminoacid linkers showed good activity against breast and glioblastoma cancer cell lines [89]. Recently dihydroartemisinin–UDCA derivatives were reported to improve the cytotoxicity of dihydroartemisinin towards leukemia cells [90] and hepatocellular carcinoma [91]. A deoxycholic acid-Camptothecin conjugate [92] was recently proved by Xiao et al. to enhance the targeted delivery of anticancer molecules in liver by exploiting the specific BA-BA receptors interaction. A series of nucleoside [93] and platinum(II) [94]-BADs were screened to test the cytotoxic activity in different tumor cell lines. A bioconjugate of 4-nitro-3-(trifluoromethyl)-aniline with UDCA was proposed by Navacchia et al. as photochemotherapeutic agent thanks to its ability to release the antimicrobial and antioxidant agent NO upon visible light input [95]. Different moieties were introduced on the BAs scaffold to inhibit the activity of Tyrosyl-DNA phosphodiesterase 1 that is an enzyme involved in removing DNA damage caused by the anticancer topoisomerase I poison drugs [96,97].

Antimicrobial activity is mainly based on the ability of BAs to damage membranes resulting from their amphiphilic steroidal structure [98]. In addition, BAs can hinder bioenergetics processes by intracellular acidification, reducing proton motive forces, DNA damaging and protein denaturation [99]. Based on the antimicrobial activity of natural BAs, BADs have been synthesized to be used as antifouling agents [100]. Moreover, a class of BA-based antibiotics termed Ceragenins have been prepared by covalently attaching amines to BAs, inspired by the molecular structure of squalamine, a naturally occurring aminosterol with potent antimicrobial activity, isolated from shark liver [101,102,103]. Similar molecules have been linked as pendants to polymers able to locally cluster the facial amphiphilicity of these cationic steroid antibiotics, thereby enhancing interactions with bacterial membranes [104,105,106] (Figure 3g–i). To date, BAs are actually used both as precursor and co-agent in drug formulation. BA-drug conjugates have been synthesized to make the drug target liver and to enhance its intestinal absorption, by exploiting the ability of the conjugate to enter the enterohepatic circulation exploiting the BA transport system. Based on these principles, BA-based nanocarriers and BADs have been synthesized to target the apical sodium-dependent BA transporter with inhibitors [107], antiviral [108] and anticancer [109] drugs. Moreover, drugs against hepatitis C virus and anticancer cytostatic drugs have been specifically targeted to the liver upon conjugation with BAs [110,111]. With the emergence of the COVID-19 pandemic, natural and synthetic BAs derivatives have also been investigated as anti-SARS-CoV2–2 agents [112,113].

## 6. BA-Based Polymers

**Figure 4:**
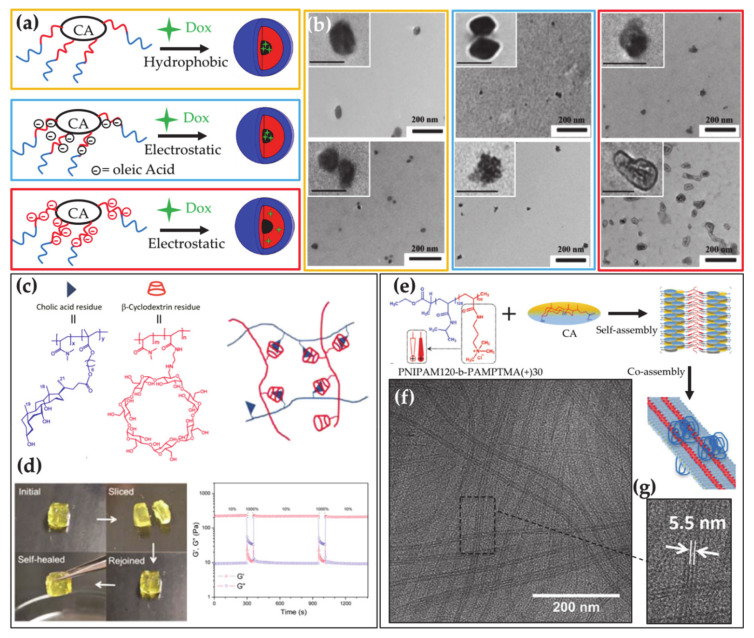
(**a**) Star-shaped block copolymers are able to load the drug Doxorubicin (Dox) through hydrophobic interaction (yellow box) or electrostatic interaction with (blue frame) or without (red frame) oleic acid (OA) as cosurfactant. (**b**) TEM micrographs of the star-polymer micelles before (upper panel) and after (lower panel) Dox loading. Adapted with permission from Cunningham, A.J.; Robinson, M.; Banquy, X.; Leblond, J.; Zhu, X.X. Bile Acid-Based Drug Delivery Systems for Enhanced Doxorubicin Encapsulation: Comparing Hydrophobic and Ionic Interactions in Drug Loading and Release *Mol. Pharm.* **2018**, *15*, 1266–1276. Ref. [118] Copyright 2018 American Chemical Society(**c**) Block-copolymer formed by cholic acid and b-cyclodextrin residues assembling into a self-healing gel; schematic representation showing the interplay among the residues in the gel matrix (**top**). (**d**) Optical (left) and rheological (right) evidence of the gel break and self-healing process (adapted with permission from Jia, Y.G.; Zhu, X.X. Self-Healing Supramolecular Hydrogel Made of Polymers Bearing Cholic Acid and β-Cyclodextrin Pendants. *Chem Mater* **2015**, *27*, 1, 387–393. [120] Copyright (2015) American Chemical Society). (**e**) The block copolymer PNIPAM120-*b*-PAMPTMA(+)30 when mixed with CA self-assembles into tape-like complexes where single stripes present recurring spacing. (**f**,**g**) Cryo TEM images of the tape-like aggregates (adapted from Schillén, K.; Galantini, L.; Du, G.; Del Giudice, A.; Alfredsson, V.; Carnerup, A.M.; Pavel, N.V.; Masci, G.; Nyström, B. Block Copolymers as Bile Salt Sequestrants: Intriguing Structures Formed in a Mixture of an Oppositely Charged Amphiphilic Block Copolymer and Bile Salt. *Phys. Chem. Chem. Phys.* **2019**, *21*, 12518–12529. Ref. [141] Published by the PCCP Owner Societies).

BAs can be used in the preparation of polymers with a main interest in drug delivery [114]. The presence of the carboxylic and hydroxyl groups on their molecule allows them to anchor polymer chains or to join into BA-based chains. BA containing polymers have been extensively investigated and recently overviewed by Zhu and co-workers [115]. According to the former approach, BAs can be used as templates onto which polymers are grown, by exploiting the hydroxyl and the carboxylic groups as junctions for polymer chains, thereby providing star polymers [116]. The star polymers self-assemble into micelles with a hollow-core [117] able to encapsulate large drug loads, which depends on the number of BA hydroxyl groups (i.e., the number of polymer branches) (Figure 4a,b). The doxorubicin-loading ability of star polymers formed by poly(allyl glycidyl ether) and poly(ethylene glycol) grafted from the CA was recently analyzed, showing that a particularly high loading is achieved by exploiting electrostatic interactions. In addition, a remarkable cellular internalization was observed for the loaded carrier [118]. BAs can be also introduced as pendant groups in block copolymers and thereby used to tune their properties and functionalities such as pH- and thermo-responsiveness and self-healing properties (Figure 4c,d) [119,120]. The investigation of the aggregates formed by these macromolecules has been promoted for their potential in drug and gene delivery applications [119,121,122,123].

Polymers formed by BAs as repeating units in the main chain, have also been synthesized with branched or linear architectures [124], mainly studied for their elastomeric and shape memory properties [125]. CA has also been introduced as pendants in the hydrophobic diblock copolymers containing a glucosamine-based hydrophilic block, for bio-related applications. It was demonstrated that the self-assembly of the block copolymer could be tuned by changing the length of the blocks to provide micelle with optimal drug loading ability [126]. Recently a block copolymer containing a dextran block linked to semi-rigid deoxycholic acid-oligo ethylene glycol polyester, was investigated, which showed a rich self-assembly involving star-shaped and wormlike micelles and vesicles depending on the dextran block length [127].

## 7. BA Lipid Mixtures

BAs and lipids can form mixed micelles exploited to solubilize hydrophobic drugs and to improve their bioavailability [50,128] BA containing liposomes, sometimes named bilosomes, can be also prepared by intercalating BAs in the liposome lecithin bilayer. Bilosomes are preferentially used to deliver oral administrated drugs and vaccines due to their ability to stand acid conditions, enzyme attack and bile salt degradation in the gastrointestinal track compared to pure liposomes. They have also been observed to increase the absorption of oral administrated insulin [129] and to facilitate transdermal drug delivery [130].

## 8. BA Polymer Mixtures

Biocompatible polymers are versatile systems widely exploited in applications. Based on their composition and sample conditions they can be available as solids, free chains, covalently crosslinked nano- and micro-gels or self-assembled nanoparticles, thus providing a platform adaptable for performances in several fields including drug delivery, tissue engineering, sensors, and catalysis. Interactions of BAs with cationic polymers are crucial for sequestration in the treatment of BA malabsorption or hypercholesterolemia. However, there is room for improvement of the currently used sequestrants of water-insoluble cationic hydrogels that have poor patient compliance [28,29] and recently some reports on new polymeric BA sequestrants have appeared in the literature [131,132]. Block copolymers able to strongly interact both electrostatically and hydrophobically with BAs have been proposed as compelling alternative sequestrants [133,134]. Investigations on the interaction between BAs and poloxamers have recently demonstrated that BAs can be adsorbed on the corona of block copolymer micelles, thereby promoting block copolymers as alternative BA sequestrants [135,136,137]. Similar Pluronic/BA mixed micelles were proven to be appealing drug-delivery vehicles and to efficiently load drugs like Clozapine [138] and Doxorubicin [139,140]. Because the BAs are anionic surfactants, it is expected that cationic block copolymers provide better BA binding and sequestration. Therefore, in a recent paper the co-assembly in dilute aqueous solution was reported in mixtures of diblock copolymer constituted of one poly(*N*-isopropyl acryl amide) (PNIPAM) block and a cationic polymer block of poly(3-acrylamidopropyl)-trimethylammonium chloride (PAMPTMA(+)) and the oppositely charged BA surfactant sodium deoxycholate. Two kinds of mixed aggregates were observed to form at room temperature having a globular morphology or a longitudinally striped tape-like architecture, which have a roughly neutral zeta potential at all compositions of the mixtures (Figure 4e–g) [141]. It was also found that the loss of water-solubility of PNIPAM with increasing temperature induces pronounced aggregation at a transition temperature, to provide aggregates with an interior containing dehydrated PNIPAM and a remarkably positive or negative charge depending on the mixture composition, thereby promoting the block copolymer–BA complex as versatile smart material for nanotechnological application. It was also demonstrated that precipitation of the complexes occurs at large fraction of BA as those encountered by sequestrants in the gastrointestinal track [142]. A thermoresponsive BAD was observed to interact with the same catanionic block copolymer to form a complex, for which an intriguing thermoresponse was revealed by scattering techniques and circular dichroism measurements [143].

Polymeric carriers for the oral administration of drugs are expected to interact with BAs, which may affect the drug solubilization and absorption. With this motivation, the interaction in mixtures of sodium taurocholate and widely used hydrophilic model polymers such as hydroxypropyl methylcellulose and polyvinylpyrrolidone, was investigated, revealing that the formation of mixed BA/polymer aggregates occurs in the mixtures, which could significantly affect the drug solubilization in the gut when hydrophilic polymers are used as dispersant [144].

## 9. Self-Assembly of Natural and Chemically Modified BAs

BA salts, here simply referred to as BAs, are soluble in water and provide self-assembly. Unlike conventional surfactants with the typical head–tail amphiphilic structure these salts have a rigid molecular structure with, in the majority of the cases, a well-defined facial amphiphilicity, resulting in a more complex self-assembly. As a matter of fact, it is well established that aggregation of BAs is driven by the interplay of hydrophobic interactions and hydrogen bonds involving hydroxyl, amide and carboxylic groups and provide aggregates with merged hydrophobic and the hydrophilic domains. The issue is still debated among several self-assembly models including the stepwise mechanism from globular primary micelles to secondary elongated ones [145,146], micellar disks [147] or helical aggregates [148,149].

In water, BAs form micelles with low aggregation number (2–20) in an associated process that is often observed to be gradual and without a sharp critical micellar concentration. Reported critical micellar concentrations decrease relative to a decrease in the number of hydroxyl groups (increasing hydrophobicity) according with the order cholate < deoxycholate [150,151,152], but also strongly depend on their positions and orientations with ursocholate and ursodeoxycholate that have hydroxyl groups in *β*-orientation presenting a lower cmc than the cholate and deoxycholate.

A decrease in the repulsions among the charged heads of deprotonated BAs can be induced by increasing the ionic strength or by decreasing the pH (for carboxylate BAs), thus promoting unidirectional growth of the micelles. Fibrils form because of the growth that can lead to gelation. For example, it is well known that DCA forms gels in water upon lowering pH around neutrality [4,153], with specific features induced by the used acid [154] or the presence of additives [155,156]. Gelation is also promoted by increasing the electrolyte concentration in DC, glycodeoxycholic (GDCA), LCA, and taurodeoxycholic (TDCA) acid salt solutions [149,157]. In such gels, the chirality of the building blocks is extended also at supramolecular level [158] where the helical structures of the fibrils, analogous to those observed in some BA crystal, [148,149,159], are observed.

Multivalent cations like Ca^2+^ can crosslink BA charge heads promoting micellar growth and gelation for cholate and GDCA. Crosslinking and formation of nanohelices can be induced on CA solution also by other multivalent cations, like those of transition metals [160,161] and lanthanides [162,163,164]. Similarly, gels can be formed by LCA upon addition of alkaline earth, lanthanide, and transition-metal ions, [165] and by DCA in the presence of europium nitrate [166].

Electrolyte-induced wormlike micelle growth and gelation is also observed in lecithin/BA aqueous mixtures [167,168]. A similar growth, but with a different mechanism is observed in lecithin/BA mixtures in oil, where reverse micelles of lecithin turn wormlike upon inclusion of the BAs in their interior [169,170].

**Figure 5:**
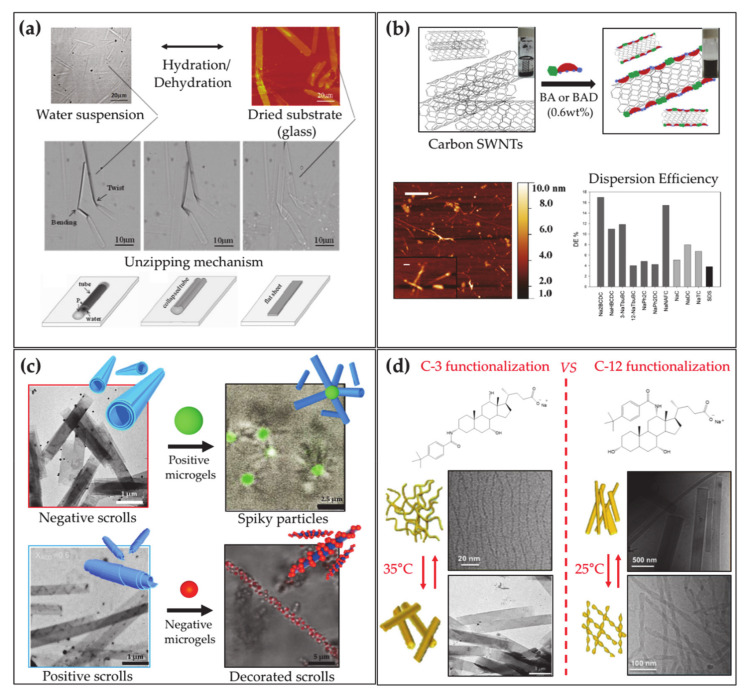
(**a**) Co-assembled lithocholate/taurolithocholate tubes (optical microscopy image, top left) are longitudinally unzipped into flat structures (Atomic Force Microscopy image, top right) by capillary force upon dehydration on substrates. The process of unzipping is proved by optical microscopy images (center) and schematized (bottom) (adapted with permission from Zhang, X.; Bera, T.; Liang, W.; Fang, J. Longitudinal Zipping/Unzipping of Self-Assembled Organic Tubes *J. Phys. Chem. B*, **2011**, *115*, 14445–14449. [175] Copyright (2011) American Chemical Society). (**b**) BA or BA derivatives facially interact with carbon nanotubes’ surface, enabling their dispersion in water solution (scheme, top). Atomic Force Microscopy images of BA derivative dispersed carbon nanotubes in water (bottom right). Graph reporting the carbon nanotubes dispersion efficiency upon addition of BA derivatives, natural BA and conventional head–tail surfactant SDS (dark grey, light grey, black bars, respectively, bottom right) (adapted with permission from Gubitosi, M.; Trillo, J.V.; Alfaro Vargas, A.; Pavel, N.V.; Gazzoli, D.; Sennato, S.; Jover, A.; Meijide, F.; Galantini, L. Characterization of Carbon Nanotube Dispersions in Solutions of Bile Salts and Derivatives Containing Aromatic Substituents *J. Phys. Chem. B* **2014**, *118*, 1012–1021. [181] Copyright (2014) American Chemical Society). (**c**) BA derivative-based scrolls having negative (top left, red frame) and positive charge (bottom left, blue frame) interact with positive and negatively charged microgels, respectively, giving rise to electrostatically stabilized supracolloidal aggregates (right) (from Cautela, J.; Lattanzi, V.; Månsson, L.; Galantini, L.; Crassous, J.J. Sphere–Tubule Superstructures through Supramolecular and Supracolloidal Assembly Pathways *Small* **2018**, *14*, 1803215. [199] Copyright (2018) Wiley). (**d**) Thermoresponsive cholic acid derivatives functionalized with a tert-butyl phenyl amide residue on C-3 (top left) and C-12 (top right). Microscopy images of the structures forming at lower and higher temperatures than the critical transition temperatures for the C-3 (bottom left) and C-12 (bottom right) derivatives (from Galantini, L.; Leggio, C.; Jover, A.; Meijide, F.; Pavel, N.V.; Soto Tellini, V.H.; Vázquez Tato, J.; Di Leonardo, R.; Ruocco, G. Kinetics of Formation of Supramolecular Tubules of a Sodium Cholate Derivative. *Soft Matter* **2009**, *5*, 3018–3025. [184] permission conveyed through Copyright Clearance Center, Inc. Cautela, J.; Severoni, E.; Redondo-Gómez, C.; di Gregorio, M.C.; Del Giudice, A.; Sennato, S.; Angelini, R.; D’Abramo, M.; Schillén, K.; Galantini, L. C-12 vs. C-3 Substituted Bile Salts: An Example of the Effects of Substituent Position and Orientation on the Self-Assembly of Steroid Surfactant Isomers. *Colloids Surf. B.* **2020**, *185*, 110556. [201] Copyright (2019), with permission from Elsevier.

The various self-assembly behavior of natural BA includes the formation of tubular aggregates, provided by LCA at strong alkaline conditions (pH = 12) [171,172]. It was shown by Fang and coworkers that pH variation can induce a switch of the tubules from spiral to a straight shape by changing pH [173]. The same authors demonstrated that LCA can form ribbon-like J or tubular H aggregates when mixed with cyanine dyes [174], and mixed tubules with TLC that can reversibly open/close by controlling the hydration (Figure 5a) [175].

It is important to remark that BAs are chiral molecules, and can selectively interact with enantiomers of additives both in monomeric and micellar form. This ability was reported for the interaction of monomers and micelles of DCA [176], GCA, and TCA [177] with the enantiomeric conformers of bilirubin-IXa, and recently for CA and DCA with binaphthyl enantiomers [178]. BA micellar self-assembly was extensively investigated in the past and exhaustively described in a recent review [4]. In addition, it is worthy to stress that, in the light of the use of BAs in the formulation of drugs, the effect of active molecules on the self-assembly of BAs, as recently reported for CA and DCA in the presence of the antibiotic drug ceftriaxone, is a particularly relevant topic [179].

Beside the drug-conjugated derivatives employed in medicine and the polymeric molecules, a broad family of molecules can be synthesized starting from BAs, to provide an expanded set of self-assembling biomaterials [6]. Derivatives can be prepared with dispersing ability of diverse materials like proteins [180] or carbon nanotubes (Figure 5b) [181]. In addition, BAs can be used as substrates to prepare an ensemble of steroidal building blocks for the fabrication of unconventional supramolecular nanostructures. Typically, fibers or ribbons are formed together with more complex tubular nano- and micro-aggregates with diameters ranging from a few nanometers to half a micron. Tubules are reported to form in aqueous systems of C-3 substituted BA with various residues such as aromatic organic groups [6,182,183,184,185,186,187,188] or amino acids [189,190,191,192], and sugars [193,194], often via stimuli-responsive self-assembly, triggered by pH [182,183] or temperature [184,195]. Interesting systems of tubules with tunable charge or diameter have been implemented by mixtures of cationic and anionic derivatives [196,197] or precursor and derivative [198]. In addition, it was recently demonstrated that BA derivative tubules are suitable elements for higher order self-assembly, providing supracolloidal non-obvious aggregates when mixed with microgels, with relevant potential in the preparation of new functional materials (Figure 5c) [199].

Keeping the same substituent, the ability to form tubules was reported to be lost for aminoacid substituted derivatives obtained from more hydrophilic BA precursors [191,192], whereas for organic aromatic substituent, tubules with remarkably different sizes were observed to form by BA differing for number, positions and orientations of the hydroxyl groups [184,185,186,195]. Spectroscopies, such as circular dichroism, show that the substituents are strongly involved in intermolecular interactions in the aggregates thereby demonstrating their relevance in the self-assembly of the derivatives. The ability to form tubules in sugar substituted derivatives is lost when glucose instead of mannose [200] is used as substituent, thereby remarking the key role of the substituent and its specific interactions in the aggregation. Recently, selectively C-3 and C-12 substituted CA derivatives were investigated, highlighting crucial effects of position and orientation of the substituent on the self-assembly: both systems showed thermoresponsive assembly at similar critical temperature, probably due to the nature of the substituent, but involving morphologically different structures according to the position and stereochemistry of the substituting residue [201] (Figure 5d). Exploiting the cross-linking action of cations [202], stable gels of helical ribbons closed into hollow cylinders have been reported to form by calcium ions and phenylalanine substituted deoxycholate at high pH [203].

Several BAs functionalized at the carboxylic groups to form different species like hydrazide [204] and aminoacid [205] esters [206] and conjugate [207,208] have also been widely investigated for their organo- or hydro-gelation properties. The importance of the hydrophobic/hydrophilic balance of the derivatives on their gelation ability was clearly illustrated. Very interestingly, the gelling ability enhancement in the mixture of cationic and anionic derivatives has also been disclosed highlighting the relevant contribution to the gelation of electrostatic attraction between the derivatives charge heads [209].

## 10. Conclusions

BAs are natural molecules ubiquitously found in vertebrates. In humans, they are produced in the hepatocytes from cholesterol modification and, through a cyclic path named enterohepatic circulation, are transported from liver to intestine, lately being transferred to the blood stream by which they are re-absorbed from the liver. The unusual amphiphilic structures allow BAs to exhibit detergent functions towards different compounds, e.g., solubilization of cholesterol, fat dietary lipids and penetration/breaking of membranes. The latter feature, expressed towards bacteria, makes BAs important antibacterial elements that are able to regulate the strength of the gut microbiota. Furthermore, BAs work in the body as hormones, being signaling molecules for the expression of genes and regulators of different metabolic paths. The physiological roles of BAs briefly summarized here and comprehensively overviewed by other specialist reports [210,211,212,213,214], opened the field for an extensive investigation of BAs and BADs use in biomedical applications including antimicrobics, anti-cholesterol drugs, regulator of dietary lipid uptake and drug carriers in co-formulations with other molecules (e.g., lipids and polymers). The application possibilities broaden even more when adopting BADs. BADs have been shown to be suitable building blocks for self-assembly structures showing a wide range of properties (e.g., gelling ability, stimuli responsiveness, self-healing). Such aggregates have been reported to be useful both in biological fields and material science. These aspects are going continuously growing, opening up unexpected scenarios in the preparation of different kinds of organic and inorganic nanomaterials [4].

## Data Availability

Not applicable.

## Abbreviations

BA	Bile acid
CA	Cholic acid
CDCA	Chenodeoxycholic acid
GCA	Glycocholic acid
TCA	Taurocholic acid
TCDCA	Taurochenodeoxycholic acid
GCDCA	Glycochenodeoxycholic acid
DCA	Deoxycholic acid
LCA	Lithocholic acid
CYP7A1	Cholesterol 7α-hydroxylase
CYP27	Sterol 27-hydroxylase
CY8B1	12α-hydroxylase
CYP7B1	Oxoysterol 7α-hydroxylase
BAD	Bile acid derivative
PNIPAM	Poly(*N*-isopropyl acryl amide)
PAMPTMA	Poly((3-acrylamido-propyl)-trimethylammonium chloride
GDCA	Glycodeoxycholic acid
TDCA	Taurodeoxycholic acid

## References

[1] Thomas C., Pellicciari R., Pruzanski M., Auwerx J., Schoonjans K. (2008). Targeting Bile-Acid Signalling for Metabolic Diseases. Nat. Rev. Drug Discov..

[2] Barbier O., Trottier J., Kaeding J., Caron P., Verreault M. (2009). Lipid-Activated Transcription Factors Control Bile Acid Glucuronidation. Mol. Cell. Biochem..

[3] Kuhajda K., Kandrac J., Kevresan S., Mikov M., Fawcett J.P. (2006). Structure and Origin of Bile Acids: An Overview. Eur. J. Drug Metab. Pharm..

[4] Madenci D., Egelhaaf S.U. (2010). Self-Assembly in Aqueous Bile Salt Solution. Curr. Opin. Colloid Interface Sci..

[5] Galantini L., di Gregorio M.C., Gubitosi M., Travaglini L., Vázquez J., Jover A., Meijide F., Soto V.H., Pavel N.V. (2015). Bile Salts and Derivatives: Rigid Unconventional Amphiphiles as Dispersants, Carriers and Superstructure Building Blocks. Curr. Opin. Colloid Interface Sci..

[6] di Gregorio M.C., Travaglini L., Del Giudice A., Cautela J., Pavel N.V., Galantini L. (2019). Bile Salts: Natural Surfactants and Precursors of a Broad Family of Complex Amphiphiles. Langmuir.

[7] Quinn R.A., Melnik A.V., Vrbanac A., Fu T., Patras K.A., Christy M.P., Bodai Z., Belda-Ferre P., Tripathi A., Chung L.K. (2020). Global Chemical Effects of the Microbiome Include New Bile-Acid Conjugations. Nature.

[8] Roberts M.S., Magnusson B.M., Burczynski F.J., Weiss M. (2002). Enterohepatic Circulation. Clin. Pharm..

[9] Russell D.W. (2009). Fifty Years of Advances in Bile Acid Synthesis and Metabolism. J. Lipid Res..

[10] Goodwin B., Jones S.A., Price R.R., Watson M.A., McKee D.D., Moore L.B., Galardi C., Wilson J.G., Lewis M.C., Roth M.E. (2000). A Regulatory Cascade of the Nuclear Receptors FXR, SHP-1, and LRH-1 Represses Bile Acid Biosynthesis. Mol. Cell.

[11] Lu T.T., Makishima M., Repa J.J., Schoonjans K., Kerr T.A., Auwerx J., Mangelsdorf D.J. (2000). Molecular Basis for Feedback Regulation of Bile Acid Synthesis by Nuclear Receptos. Mol. Cell.

[12] Denson L.A., Sturm E., Echevarria W., Zimmerman T.L., Makishima M., Mangelsdorf D.J., Karpen S.J. (2001). The Orphan Nuclear Receptor, Shp, Mediates Bile Acid-Induced Inhibition of the Rat Bile Acid Transporter, Ntcp. Gastroenterology.

[13] Kim I., Ahn S.H., Inagaki T., Choi M., Ito S., Guo G.L., Kliewer S.A., Gonzalez F.J. (2007). Differential Regulation of Bile Acid Homeostasis by the Farnesoid X Receptor in Liver and Intestine. J. Lipid Res..

[14] Kong B., Wang L., Chiang J.Y., Zhang Y., Klaassen C.D., Guo G.L. (2012). Mechanism of Tissue-Specific Farnesoid X Recptor in Suppressing the Expression of Genes in Bile-Acid Synthesis in Mice. Hepatology.

[15] Li T., Francl J.M., Boehme S., Chiang J.Y.L. (2013). Regulation of Cholesterol and Bile Acid Homeostasis by the Cholesterol 7α-Hydroxylase/Steroid Response Element-Binding Protein 2/MicroRNA-33a Axis in Mice. Hepatology.

[16] Meissner M., Wolters H., de Boer R.A., Havinga R., Boverhof R., Bloks V.W., Kuipers F., Groen A.K. (2013). Bile Acid Sequestration Normalizes Plasma Cholesterol and Reduces Atherosclerosis in Hypercholesterolemic Mice. No Additional Effect of Physical Activity. Atherosclerosis.

[17] Mazidi M., Rezaie P., Karimi E., Kengne A.P. (2017). The effects of bile acid sequestrants on lipid profile and blood glucose concentrations: A systematic review and meta-analysis of randomized controlled trials. Int. J. Cardiol..

[18] Kuipers F., Bloks V.W., Groen A.K. (2014). Beyond intestinal soap—Bile acids in metabolic control. Nat. Rev. Endocrinol..

[19] Hou R., Goldberg A.C. (2009). Lowering Low-Density Lipoprotein Cholesterol:Statins, Ezetimibe, Bile Acid Sequestrants, and Combinations: Comparative Efficacy and Safety. Endocrinol. Metab. Clin. N. Am..

[20] Sehayek E., Ono J.G., Shefer S., Nguyen L.B., Wang N., Batta A.K., Salen G., Smith J.D., Tall A.R., Breslow J.L. (1998). Biliary Cholesterol Excretion: A Novel Mechanism That Regulates Dietary Cholesterol Absorption. Proc. Natl. Acad. Sci. USA.

[21] Mandeville W., Goldberg D. (1997). The Sequestration of Bile Acids, a Non-Absorbed Method for Cholesterol Reduction: A Review. Curr. Pharm. Des..

[22] Camilleri M., Gores G.J. (2020). Therapeutic Targeting of Bile Acids. Am. J. Physiol. Gastrointest. Liver Physiol..

[23] Lundasen T., Galman C., Angelini B., Rudling M. (2006). Circulating Intestinal Fibroblast Growth Factor 19 Has a Pronounced Diurnal Variation and Modulates Hepatic Bile Acid Synthesis in Man. J. Intern. Med..

[24] Walters J.R.F., Tasleem A.L.I.M., Omer O.S., Brydon W.G., Dew T., Roux C.W.L.E. (2009). A New Mechanism for Bile Acid Diarrhea: Defective Feedback Inhibition of Bile Acid Biosynthesis. Clin. Gastroenterol. Hepatol..

[25] Johnston I., Nolan J., Pattni S.S., Walters J.R.F. (2011). New Insights into Bile Acid Malabsorption. Curr. Gastroenterol. Rep..

[26] Walters J.R.F. (2014). Bile Acid Diarrhoea and FGF19: New Views. Nat. Rev. Gastroenterol. Hepatol..

[27] Hofmann A.F., Mysels K.J. (1988). Bile Salts as Biological Surfactants. Colloids Surf..

[28] Huval C.C., Holmes-Farley S.R., Mandeville W.H., Sacchiero R., Dhal P.K. (2004). Syntheses of Hydrophobically Modified Cationic Hydrogels by Copolymerization of Alkyl Substituted Diallylamine Monomers and Their Use as Bile Acid Sequestrants. Eur. Polym. J..

[29] Wilcox C., Turner J., Green J. (2014). Systematic Review: The Management of Chronic Diarrhoea Due to Bile Acid Malabsorption. Aliment. Pharm. Ther..

[30] Hansen M., Sonne D.P., Knop F.K. (2014). Bile Acid Sequestrants: Glucose-Lowering Mechanisms and Efficacy in Type 2 Diabetes. Curr. Diab. Rep..

[31] Seetharam P., Rodrigues G. (2011). Short Bowel Syndrome: A Review of Management Options. Saudi J. Gastroenterol..

[32] Emmett M., Guirl M.J., Santa Ana C.A., Porter J.L., Neimark S., Hofmann A.F., Fordtran J.S. (2003). Conjugated Bile Acid Replacement Therapy Reduces Urinary Oxalate Excretion in Short Bowel Syndrome. Am. J. Kidney Dis..

[33] Gruy-Kapral C., Little K.H., Fordtran J.S., Meziere T.L., Hagey L.R., Hofmann A.F. (1999). Conjugated Bile ACid Replacement Theraphy for Short-Bowel Syndrome. Gastroenterology.

[34] Van de Heijning B.J.M., Stolk M.F.J., Van Erpecum K.J., Renooij W., Groen A.K., VanBerge-Henegouwen G.P. (1994). Bile Salt-Induced Cholesterol Crystal Formation from Model Bile Vesicles: A Time Course Study. J. Lipid Res..

[35] Tamesue N., Juniper K. (1967). Concentrations of Bile Salts at the Critical Micellar Concentration of Human Gall Bladder Bile. Gastroenterology.

[36] Higuchi W.I., Tzeng C.S., Chang S.J., Chiang H.J., Liu C.L. (2008). Estimation of Cholesterol Solubilization by a Mixed Micelle Binding Model in Aqueous Tauroursodeoxycholate:Lecithin:Cholesterol Solutions. J. Pharm. Sci..

[37] Reynier M.O., Montet J.C., Gerolami A., Marteau C., Crotte C., Montet A.M., Mathieu S. (1981). Comparative Effects of Cholic, Chenodeoxycholic, and Ursodeoxycholic Acids on Micellar Solubilization and Intestinal Absorption of Cholesterol. J. Lipid Res..

[38] Rudling M., Laskar A., Straniero S. (2019). Gallbladder Bile Supersaturated with Cholesterol in Gallstone Patients Preferentially Develops from Shortage of Bile Acids. J. Lipid Res..

[39] Carey M.C., Small D.M. (1978). The Physical Chemistry of Cholesterol Solubility in Bile. Relationship to Gallstone Formation and Dissolution in Man. J. Clin. Investig..

[40] Coreta-Gomes F.M., Vaz W.L.C., Wasielewski E., Geraldes C.F.G., Moreno M.J. (2016). Quantification of Cholesterol Solubilized in Dietary Micelles: Dependence on Human Bile Salt Variability and the Presence of Dietary Food Ingredients. Langmuir.

[41] Portincasa P. (2012). Therapy of Gallstone Disease: What It Was, What It Is, What It Will Be. World J. Gastrointest. Pharm. Ther..

[42] Jang S.I., Fang S., Kim K.P., Ko Y., Kim H., Oh J., Hong G.Y., Lee S.Y., Kim J.M., Noh I. (2019). Combination Treatment with N-3 Polyunsaturated Fatty Acids and Ursodeoxycholic Acid Dissolves Cholesterol Gallstones in Mice. Sci. Rep..

[43] Boyer J.L. (2013). Bile Formation and Secretion. Compr. Physiol..

[44] Funabashi M., Grove T.L., Wang M., Varma Y., McFadden M.E., Brown L.C., Guo C., Higginbottom S., Almo S.C., Fischbach M.A. (2020). A Metabolic Pathway for Bile Acid Dehydroxylation by the Gut Microbiome. Nature.

[45] Maldonado-Valderrama J., Wilde P., MacIerzanka A., MacKie A. (2011). The Role of Bile Salts in Digestion. Adv. Colloid Interface Sci..

[46] Macierzanka A., Torcello-Gómez A., Jungnickel C., Maldonado-Valderrama J. (2019). Bile Salts in Digestion and Transport of Lipids. Adv. Colloid Interface Sci..

[47] Naso J.N., Bellesi F.A., Pizones Ruiz-Henestrosa V.M., Pilosof A.M.R. (2019). Studies on the Interactions between Bile Salts and Food Emulsifiers under in Vitro Duodenal Digestion Conditions to Evaluate Their Bile Salt Binding Potential. Colloids Surf. B..

[48] Cremers C.M., Knoefler D., Vitvitsky V., Banerjee R., Jakob U. (2014). Bile Salts Act as Effective Protein-Unfolding Agents and Instigators of Disulfide Stress in Vivo. Proc. Natl. Acad. Sci. USA.

[49] Pabois O., Lorenz C.D., Harvey R.D., Grillo I., Grundy M.M.L., Wilde P.J., Gerelli Y., Dreiss C.A. (2019). Molecular Insights into the Behaviour of Bile Salts at Interfaces: A Key to Their Role in Lipid Digestion. J. Colloid Interface Sci..

[50] Moghimipour E., Ameri A., Handali S. (2015). Absorption-Enhancing Effects of Bile Salts. Molecules.

[51] Naumann S., Schweiggert-Weisz U., Eglmeier J., Haller D., Eisner P. (2019). In Vitro Interactions of Dietary Fibre Enriched Food Ingredients with Primary and Secondary Bile Acids. Nutrients.

[52] Shokry D.S., Waters L.J., Parkes G.M.B., Mitchell J.C., Snowden M.J. (2019). Formation of a Bile Salt-Drug Hydrogel to Predict Human Intestinal Absorption. J. Pharm. Sci..

[53] Torcello-Gómez A., Maldonado-Valderrama J., Martín-Rodríguez A., McClements D.J. (2011). Physicochemical Properties and Digestibility of Emulsified Lipids in Simulated Intestinal Fluids: Influence of Interfacial Characteristics. Soft Matter.

[54] Hofmann A.F., Eckmann L. (2006). How Bile Acids Confer Gut Mucosal Protection against Bacteria. Proc. Natl. Acad. Sci. USA.

[55] Begley M., Gahan C.G.M., Hill C. (2005). The Interaction between Bacteria and Bile. FEMS Microbiol. Rev..

[56] Pazzi P., Puviani A.C., Libera M.D., Guerra G., Ricci D., Gullini S., Ottolenghi C. (1997). Bile Salt-Induced Cytotoxicity and Ursodeoxycholate Cytoprotection: In-Vitro Study in Perifused Rat Hepatocytes. Eur. J. Gastroenterol. Hepatol..

[57] Sung J.Y., Shaffer E.A., Costerton J.W. (1993). Antibacterial Activity of Bile Salts against Common Biliary Pathogens—Effects of Hydrophobicity of the Molecule and in the Presence of Phospholipids. Dig. Dis. Sci..

[58] Leverrier P., Dimova D., Pichereau V., Auffray Y., Boyaval P., Jan G. (2003). Susceptibility and Adaptive Response to Bile Salts in Propionibacterium Freudenreichii: Physiological and Proteomic Anlysis. Appl. Environ. Microbiol..

[59] Fujisawa T., Mori M. (1997). Influence of Various Bile Salts on β-Glucuronidase Activity of Intestinal Bacteria. Lett. Appl. Microbiol..

[60] Coleman R., Lowe P.J., Billington D. (1980). Membrane Lipid Composition and Susceptibility to Bile Salt Damage. BBA Biomembr..

[61] Kociubinski G., Gómez Zavaglia A., Pérez P.F., Disalvo E.A., De Antoni G.L. (2002). Effect of Bile Components on the Surface Properties of Bifidobacteria. J. Dairy Res..

[62] Tian Y., Gui W., Koo I., Smith P.B., Allman E.L., Nichols R.G., Rimal B., Cai J., Liu Q., Patterson A.D. (2020). The Microbiome Modulating Activity of Bile Acids. Gut Microbes.

[63] Small D.M., Lilly H.S., Hamilton J.A., Cabral D.J. (1987). Transbilayer Movement of Bile Acids in Model Membranes. Biochemistry.

[64] Inagaki T., Moschetta A., Lee Y.K., Peng L., Zhao G., Downes M., Yu R.T., Shelton J.M., Richardson J.A., Repa J.J. (2006). Regulation of Antibacterial Defense in the Small Intestine by the Nuclear Bile Acid Receptor. Proc. Natl. Acad. Sci. USA.

[65] Kang J.D., Myers C.J., Harris S.C., Kakiyama G., Lee I.K., Yun B.S., Matsuzaki K., Furukawa M., Min H.K., Bajaj J.S. (2019). Bile Acid 7α-Dehydroxylating Gut Bacteria Secrete Antibiotics That Inhibit Clostridium Difficile: Role of Secondary Bile Acids. Cell Chem. Biol..

[66] Ma C., Han M., Heinrich B., Fu Q., Zhang Q., Sandhu M., Agdashian D., Terabe M., Berzofsky J.A., Fako V. (2018). Gut Microbiome–Mediated Bile Acid Metabolism Regulates Liver Cancer via NKT Cells. Science.

[67] Mao J., Chen X., Wang C., Li W., Li J. (2020). Effects and Mechanism of the Bile Acid (Farnesoid X) Receptor on the Wnt/β-Catenin Signaling Pathway in Colon Cancer. Oncol. Lett..

[68] Di Ciaula A., Wang D.Q.H., Molina E.M., Baccetto R.L., Calamita G., Palmieri V.O., Portincasa P. (2017). Bile Acids and Cancer: Direct and Environmental-Dependent Effects. Ann. Hepatol..

[69] Pavlović N., Goločorbin-Kon S., Danić M., Stanimirov B., Al-Salami H., Stankov K., Mikov M. (2018). Bile Acids and Their Derivatives as Potential Modifiers of Drug Release and Pharmacokinetic Profiles. Front. Pharm..

[70] Wang D.Q.H., Carey M.C. (2014). Therapeutic Uses of Animal Biles in Traditional Chinese Medicine: An Ethnopharmacological, Biophysical Chemical and Medicinal Review. World J. Gastroenterol..

[71] Goldberg A.A., Beach A., Davies G.F., Harkness T.A.A., LeBlanc A., Titorenko V.I. (2011). Lithocholic Bile Acid Selectively Kills Neuroblastoma Cells, While Sparing Normal Neuronal Cells. Oncotarget.

[72] Luu T.H., Bard J.M., Carbonnelle D., Chaillou C., Huvelin J.M., Bobin-Dubigeon C., Nazih H. (2018). Lithocholic Bile Acid Inhibits Lipogenesis and Induces Apoptosis in Breast Cancer Cells. Cell. Oncol..

[73] Goldberg A.A., Titorenko V.I., Beach A., Thomas Sanderson J. (2013). Bile Acids Induce Apoptosis Selectively in Androgen-Dependent and -Independent Prostate Cancer Cells. PeerJ.

[74] Trah J., Arand J., Oh J., Pagerols-Raluy L., Trochimiuk M., Appl B., Heidelbach H., Vincent D., Saleem M.A., Reinshagen K. (2020). Lithocholic Bile Acid Induces Apoptosis in Human Nephroblastoma Cells: A Non-Selective Treatment Option. Sci. Rep..

[75] Goossens J.F., Bailly C. (2019). Ursodeoxycholic Acid and Cancer: From Chemoprevention to Chemotherapy. Pharm. Ther..

[76] Blanchet M., Brunel J.M. (2018). Bile Acid Derivatives: From Old Molecules to a New Potent Therapeutic Use: An Overview. Curr. Med..

[77] Suh H., Jung E.J., Kim T.H., Lee H.Y., Park Y.H., Kim K.W. (1997). Anti-angiogenic activity of ursodeoxycholic acid and its derivatives. Cancer Lett..

[78] Liu H., Qin C.K., Han G.Q., Xu H.W., Ren W.H., Qin C.Y. (2008). Synthetic chenodeoxycholic acid derivative, HS-1200, induces apoptosis of human hepatoma cells via a mitochondrial pathway. Cancer Lett..

[79] Park S.E., Lee S.W., Hossain M.A., Kim M.Y., Kim M.N., Ahn E.Y., Park Y.C., Suh H., Kim G.Y., Choi Y.H. (2008). A chenodeoxycholic derivative, HS-1200, induces apoptosis and cell cycle modulation via Egr-1 gene expression control on human hepatoma cells. Cancer Lett..

[80] Im E., Choi Y.H., Paik K.J., Suh H., Jin Y., Kim K.W., Yoo Y.H., Kim N.D. (2001). Novel bile acid derivatives induce apoptosis via a p53-independent pathway in human breast carcinoma cells. Cancer Lett..

[81] Im E.-O., Lee S., Suh H., Kim K.-W., Bae Y.T., Kim N.D. (1999). A novel ursodeoxycholic acid derivative induces apoptosis in human MCF-7 breast cancer cells. Pharm. Pharmacol. Commun..

[82] Kim N.D., Im E.-O., Hyun Yoo Y., Hyun Choi Y. (2006). Modulation of the cell cycle and induction of apoptosis in human cancer cells by synthetic bile acids. Curr. Cancer Drug Targets.

[83] Choi Y.H., Im E.O., Suh H., Jin Y., Yoo Y.H., Kim N.D. (2003). Apoptosis and modulation of cell cycle control by synthetic derivatives of ursodeoxycholic acid and chenodeoxycholic acid in human prostate cancer cells. Cancer Lett..

[84] Park S., Choi H.J., Yee S.B., Chung H.Y., Suh H., Choi Y.H., Yoo Y.H., Kim N.D. (2004). Synthetic bile acid derivatives inhibit cell proliferation and induce apoptosis in HT-29 human colon cancer cells. Int. J. Oncol..

[85] Brossard D., El Kihal L., Clément M., Sebbahi W., Khalid M., Roussakis C., Rault S. (2010). Synthesis of bile acid derivatives and in vitro cytotoxic activity with pro-apoptotic process on multiple myeloma (KMS-11), glioblastoma multiforme (GBM), and colonic carcinoma (HCT-116) human cell lines. Eur. J. Med. Chem..

[86] Park K., Kim Y.S., Lee G.Y., Nam J.O., Lee S.K., Park R.W., Kim S.Y., Kim I.S., Byun Y. (2006). Antiangiogenic effect of bile acid acylated heparin derivative. Pharm. Res..

[87] Park K., Lee S.K., Son D.H., Park S.A., Kim K., Chang H.W., Jeong E.J., Park R.W., Kim I.S., Kwon I.C. (2007). The attenuation of experimental lung metastasis by a bile acid acylated-heparin derivative. Biomaterials.

[88] Singh M., Singh A., Kundu S., Bansal S., Bajaj A. (2013). Deciphering the role of charge, hydration, and hydrophobicity for cytotoxic activities and membrane interactions of bile acid based facial amphiphiles. Biochim. Biophys. Acta Biomembr..

[89] Agarwal D.S., Shilpa Anantaraju H., Sriram D., Yogees-wari P., Nanjegowda S.H., Mallu P., Sakhuja R. (2016). Synthesis, characterization and biological evaluation of bile acid-aromatic/heteroaromatic amides linked via amino acids as anti-cancer agents. Steroids.

[90] Marchesi E., Chinaglia N., Capobianco M.L., Marchetti P., Huang T.E., Weng H.C., Guh J.H., Hsu L.C., Perrone D., Navacchia M.L. (2019). Dihydroartemisinin–Bile Acid Hybridization as an Effective Approach to Enhance Dihydroartemisinin Anticancer Activity. ChemMedChem.

[91] Huang T., Deng Y., Hsu J., Leu W., Marchesi E., Perrone D., Hsu L. (2020). Evaluation of the Anticancer Activity of a Bile Acid-Dihydroartemisinin Hybrid in Hepatocellular Carcinoma Cells. 2020, 11, 1–14. Front. Pharmacol..

[92] Xiao L., Yu E., Yue H., Li Q. (2019). Enhanced Liver Targeting of Camptothecin via Conjugation with Deoxycholic Acid. Molecules.

[93] Navacchia M.L., Marchesi E., Mari L., Chinaglia N., Gallerani E., Gavioli R., Capobianco M.L., Perrone D. (2017). Rational Design of Nucleoside-Bile Acid Conjugates Incorporating a Triazole Moiety for Anticancer Evaluation and SAR Exploration. Molecules.

[94] Seroka B., Łotowski Z., Hryniewicka A., Rárová L., Sicinski R.R., Tomkiel A.M., Morzycki J.W. (2020). Synthesis of New Cisplatin Derivatives from Bile Acids. Molecules.

[95] Navacchia M.L., Fraix A., Chinaglia N., Gallerani E., Perrone D., Cardile V., Graziano A.C.E., Capobianco M.L., Sortino S. (2016). NO Photoreleaser-Deoxyadenosine and -Bile Acid Derivative Bioconjugates as Novel Potential Photochemotherapeutics. ACS Med. Chem. Lett..

[96] Salomatina O.V., Popadyuk I.I., Zakharenko A.L., Olga D., Chepanova A.A., Dyrkheeva N.S., Komarova N.I., Reynisson J., Anarbaev R.O., Salakhutdinov N.F. (2020). Deoxycholic Acid as a Molecular Scaffold for Tyrosyl-DNA Phosphodiesterase 1 Inhibition: A Synthesis, Structure-Activity Relationship and Molecular Modeling Study. Steroids.

[97] Salomatina O.V., Popadyuk I.I., Zakharenko A.L., Zakharova O.D., Fadeev D.S., Komarova N.I., Reynisson J., Arabshahi H.J., Chand R., Volcho K.P. (2018). Novel Semisynthetic Derivatives of Bile Acids as Effective Tyrosyl-DNA Phosphodiesterase 1 Inhibitors. Molecules.

[98] Schölmerich J., Becher M.-S., Schmidt K., Schubert R., Kremer B., Feldhaus S., Gerok W. (1984). Influence of Hydroxylation and Conjugation of Bile Salts on Their Membrane-Damaging Properties-Studies on Isolated Hepatocytes and Lipid Membrane Vesicles. Hepatology.

[99] Sannasiddappa T.H., Lund P.A., Clarke S.R. (2017). In Vitro Antibacterial Activity of Unconjugated and Conjugated Bile Salts on Staphylococcus Aureus. Front. Microbiol..

[100] Neves A.R., Almeida J.R., Carvalhal F., Câmara A., Pereira S., Antunes J., Vasconcelos V., Pinto M., Silva E.R., Sousa E. (2020). Overcoming Environmental Problems of Biocides: Synthetic Bile Acid Derivatives as a Sustainable Alternative. Ecotoxicol. Environ. Saf..

[101] Guan Q., Li C., Schmidt E.J., Boswell J.S., Walsh J.P., Allman G.W., Savage P.B. (2000). Preparation and Characterization of Cholic Acid-Derived Antimicrobial Agents with Controlled Stabilities. Org. Lett..

[102] Ding B., Guan Q., Walsh J.P., Boswell J.S., Winter T.W., Winter E.S., Boyd S.S., Li C., Savage P.B. (2002). Correlation of the Antibacterial Activities of Cationic Peptide Antibiotics and Cationic Steroid Antibiotics. J. Med. Chem..

[103] Savage P.B., Li C., Taotafa U., Ding B., Guan Q. (2002). Antibacterial Properties of Cationic Steroid Antibiotics. FEMS Microbiol. Lett..

[104] Rahman M.A., Sha Y., Jui M.S., Lamm M.E., Ma Y., Tang C. (2019). Facial Amphiphilicity-Induced Self-Assembly (FAISA) of Amphiphilic Copolymers. Macromolecules.

[105] Rahman M.A., Jui M.S., Bam M., Cha Y., Luat E., Alabresm A., Nagarkatti M., Decho A.W., Tang C. (2020). Facial Amphiphilicity-Induced Polymer Nanostructures for Antimicrobial Applications. ACS Appl. Mater. Interfaces.

[106] Rahman M.A., Bam M., Luat E., Jui M.S., Ganewatta M.S., Shokfai T., Nagarkatti M., Decho A.W., Tang C. (2018). Macromolecular-Clustered Facial Amphiphilic Antimicrobials. Nat. Commun..

[107] Kramer W., Glombik H. (2006). Bile Acid Reabsorption Inhibitors (BARI): Novel Hypolipidemic Drugs. Curr. Med. Chem..

[108] Tolle-Sander S., Lentz K.A., Maeda D.Y., Coop A., Polli J.E. (2004). Increased Acyclovir Oral Bioavailability via a Bile Acid Conjugate. Mol. Pharm..

[109] Jha S.K., Chung J.Y., Pangeni R., Choi H.S., Subedi L., Kweon S., Choi J.U., Byun Y., Kim Y.H., Park J.W. (2020). Enhanced Antitumor Efficacy of Bile Acid-Lipid Complex-Anchored Docetaxel Nanoemulsion via Oral Metronomic Scheduling. J. Control. Release.

[110] Sievänen E. (2007). Exploitation of Bile Acid Transport Systems in Prodrug Design. Molecules.

[111] Kramer W. (2011). Transporters, Trojan Horses and Therapeutics: Suitability of Bile Acid and Peptide Transporters for Drug Delivery. Biol. Chem..

[112] Subramanian S., Iles T., Ikramuddin S., Steer C.J. (2020). Merit of an Ursodeoxycholic Acid Clinical Trial in COVID-19 Patients. Vaccines.

[113] Carino A., Moraca F., Fiorillo B., Marchianò S., Sepe V., Biagioli M., Finamore C., Bozza S., Francisci D., Distrutti E. (2020). Hijacking SARS-CoV-2/ACE2 Receptor Interaction by Natural and Semi-Synthetic Steroidal Agents Acting on Functional Pockets on the Receptor Binding Domain. Front. Chem..

[114] Zhao Y., Cho H., Widanapathirana L., Zhang S. (2013). Conformationally Controlled Oligocholate Membrane Transporters: Learning through Water Play. Acc. Chem. Res..

[115] Le Dévédec F., Fuentealba D., Strandman S., Bohne C., Zhu X.X. (2012). Aggregation Behavior of Pegylated Bile Acid Derivatives. Langmuir.

[116] Giguère G., Zhu X.X. (2010). Functional Star Polymers with a Cholic Acid Core and Their Thermosensitive Properties. Biomacromolecules.

[117] Despa F., Luo J.T., Li J., Duan Y., Lam K.S. (2010). Cholic Acid Micelles—Controlling the Size of the Aqueous Cavity by PEGylation. Phys. Chem. Chem. Phys..

[118] Cunningham A.J., Robinson M., Banquy X., Leblond J., Zhu X.X. (2018). Bile Acid-Based Drug Delivery Systems for Enhanced Doxorubicin Encapsulation: Comparing Hydrophobic and Ionic Interactions in Drug Loading and Release. Mol. Pharm..

[119] Pal S., Ghosh Roy S., De P. (2014). Synthesis via RAFT Polymerization of Thermo- and PH-Responsive Random Copolymers Containing Cholic Acid Moieties and Their Self-Assembly in Water. Polym. Chem..

[120] Jia Y.G., Zhu X.X. (2015). Self-Healing Supramolecular Hydrogel Made of Polymers Bearing Cholic Acid and β-Cyclodextrin Pendants. Chem. Mater..

[121] Shao Y., Jia Y.G., Shi C., Luo J., Zhu X.X. (2014). Block and Random Copolymers Bearing Cholic Acid and Oligo(Ethylene Glycol) Pendant Groups: Aggregation, Thermosensitivity, and Drug Loading. Biomacromolecules.

[122] Du J., Tang Y., Lewis A.L., Armes S.P. (2005). PH-Sensitive Vesicles Based on a Biocompatible Zwitterionic Diblock Copolymer. J. Am. Chem. Soc..

[123] Xiong Y., Qi J., Yao P. (2012). Amphiphilic Cholic-Acid-Modified Dextran Sulfate and Its Application for the Controlled Delivery of Superoxide Dismutase. Macromol. Biosci..

[124] Ahlheirn M., Hallensleben M.L. (1988). Kondensationspolymerisation von Gallensäuren Herrn. 1988, 9, 299–302. Macromol. Rapid Commun..

[125] Shao Y., Lavigueur C., Zhu X.X. (2012). Multishape Memory Effect of Norbornene-Based Copolymers with Cholic Acid Pendant Groups. Macromolecules.

[126] Zhang K., Jia Y.G., Tsai I.H., Strandman S., Ren L., Hong L., Zhang G., Guan Y., Zhang Y., Zhu X.X. (2017). “Bitter-Sweet” Polymeric Micelles Formed by Block Copolymers from Glucosamine and Cholic Acid. Biomacromolecules.

[127] Nichifor M., Stanciu M.C., Doroftei F. (2021). Self-Assembly of Dextran–b–Deoxycholic Acid Polyester Copolymers: Copolymer Composition and Self-Assembly Procedure Tune the Aggregate Size and Morphology. Carbohydr. Polym..

[128] Stojančević M., Pavlović N., Goločorbin-Kon S., Mikov M. (2013). Application of Bile Acids in Drug Formulation and Delivery. Front. Life Sci..

[129] Damgé C.P., Maincent P. (2008). Nanoparticles Strategies for the Oral Delivery of Insulin. Expert Opin. Drug Deliv..

[130] Al-Mahallawi A.M., Abdelbary A.A., Aburahma M.H. (2015). Investigating the Potential of Employing Bilosomes as a Novel Vesicular Carrier for Transdermal Delivery of Tenoxicam. Int. J. Pharm..

[131] Mendonça P.V., Serra A.C., Silva C.L., Simões S., Coelho J.F.J. (2013). Polymeric Bile Acid Sequestrants—Synthesis Using Conventional Methods and New Approaches Based on “Controlled”/Living Radical Polymerization. Prog. Polym. Sci..

[132] Heřmánková E., Žák A., Poláková L., Hobzová R., Hromádka R., Širc J. (2018). Polymeric Bile Acid Sequestrants: Review of Design, in Vitro Binding Activities, and Hypocholesterolemic Effects. Eur. J. Med. Chem..

[133] Cameron N.S., Eisenberg A., Brown G.R. (2002). Amphiphilic Block Copolymers as Bile Acid Sorbents: 1. Synthesis of Polystyrene-b-Poly(N,N,N-Trimethylammoniumethylene Acrylamide Chloride). Biomacromolecules.

[134] Cameron N.S., Eisenberg A., Brown G.R. (2002). Amphiphilic Block Copolymers as Bile Acid Sorbents: 2. Polystyrene-b-Poly(N,N,N-Trimethylammoniumethylene Acrylamide Chloride): Self-Assembly and Application to Serum Cholesterol Reduction. Biomacromolecules.

[135] Bayati S., Galantini L., Knudsen K.D., Schillén K. (2015). Effects of Bile Salt Sodium Glycodeoxycholate on the Self-Assembly of PEO-PPO-PEO Triblock Copolymer P123 in Aqueous Solution. Langmuir.

[136] Bayati S., Anderberg Haglund C., Pavel N.V., Galantini L., Schillén K. (2016). Interaction between Bile Salt Sodium Glycodeoxycholate and PEO-PPO-PEO Triblock Copolymers in Aqueous Solution. RSC Adv..

[137] Patel V., Ray D., Bahadur A., Ma J., Aswal V.K., Bahadur P. (2018). Pluronic^®^-Bile Salt Mixed Micelles. Colloids Surf. B..

[138] Singla P., Singh O., Chabba S., Aswal V.K., Mahajan R.K. (2018). Sodium Deoxycholate Mediated Enhanced Solubilization and Stability of Hydrophobic Drug Clozapine in Pluronic Micelles. Spectrochim. Acta Part A Mol. Biomol. Spectrosc..

[139] Tasca E., Giudice A. Del, Galantini L., Schillén K., Giuliani A.M., Giustini M. (2019). A Fluorescence Study of the Loading and Time Stability of Doxorubicin in Sodium Cholate/PEO-PPO-PEO Triblock Copolymer Mixed Micelles. J. Colloid Interface Sci..

[140] Tasca E., Andreozzi P., Del Giudice A., Galantini L., Schillén K., Giuliani A.M., de los Angeles Ramirez M., Moya S.E., Giustini M. (2020). Poloxamer/Sodium Cholate Co-Formulation for Micellar Encapsulation of Doxorubicin with High Efficiency for Intracellular Delivery: An in-Vitro Bioavailability Study. J. Colloid Interface Sci..

[141] Schillén K., Galantini L., Du G., Del Giudice A., Alfredsson V., Carnerup A.M., Pavel N.V., Masci G., Nyström B. (2019). Block Copolymers as Bile Salt Sequestrants: Intriguing Structures Formed in a Mixture of an Oppositely Charged Amphiphilic Block Copolymer and Bile Salt. Phys. Chem. Chem. Phys..

[142] Du G., Del Giudice A., Alfredsson V., Carnerup A.M., Pavel N.V., Loh W., Masci G., Nyström B., Galantini L., Schillén K. (2020). Effect of Temperature on the Association Behavior in Aqueous Mixtures of an Oppositely Charged Amphiphilic Block Copolymer and Bile Salt. Polymer.

[143] di Gregorio M.C., Gubitosi M., Travaglini L., Pavel N.V., Jover A., Meijide F., Vázquez Tato J., Sennato S., Schillén K., Tranchini F. (2017). Supramolecular Assembly of a Thermoresponsive Steroidal Surfactant with an Oppositely Charged Thermoresponsive Block Copolymer. Phys. Chem. Chem. Phys..

[144] Pigliacelli C., Belton P., Wilde P., Qi S. (2019). Probing the Molecular Interactions between Pharmaceutical Polymeric Carriers and Bile Salts in Simulated Gastrointestinal Fluids Using NMR Spectroscopy. J. Colloid Interface Sci..

[145] Carey M.C., Small D.M. (1969). Micellar Properties of Dihydroxy and Trihydroxy Bile Salts: Effects of Counterion and Temperature. J. Colloid Interface Sci..

[146] Li Y., Holzwarth J.F., Bohne C. (2000). Aggregation Dynamics of Sodium Taurodeoxycholate and Sodium Deoxycholate. Langmuir.

[147] Kawamura H., Murata Y., Yamaguchi T., Igimi H., Tanaka M., Sugihara G., Kratohvil J.P. (1989). Spin-Label Studies of Bile Salt Micelles. J. Phys. Chem..

[148] Campanelli A.R., de Sanctis S.C., Galantini L., Giglio E., Scaramuzza L. (1991). A Possible Helical Model for Sodium Glycocholate Micellar Aggregates. J. Incl. Phenom. Mol. Recognit. Chem..

[149] D’Archivio A.A., Galantini L., Gavuzzo E., Giglio E., Scaramuzza L. (1996). Possible Models for the Micellar Aggregates of Glycocholate and Taurocholate Salts from Crystal Structures, QELS, and CD Measurements. Langmuir.

[150] Galantini L., Giglio E., Leonelli A., Pavel N.V. (2004). An Integrated Study of Small-Angle x-Ray Scattering and Dynamic Light Scattering on Cylindrical Micelles of Sodium Glycodeoxycholate. J. Phys. Chem. B.

[151] Galantini L., Giampaolo S.M., Mannina L., Pavel N.V., Viel S. (2004). Study of Intermicellar Interactions and Micellar Sizes in Ionic Micelle Solutions by Comparing Collective Diffusion and Self-Diffusion Coefficients. J. Phys. Chem. B.

[152] D’Archivio A.A., Galantini L., Tettamanti E. (2000). Study on Intermicellar Interactions and Micellar Size in Aqueous Solutions of Sodium Taurocholate by Measurements of Collective Diffusion and Self-Diffusion Coefficients. J. Phys. Chem. B.

[153] Zhang J., Wang H., Li X., Song S., Song A., Hao J. (2016). Two Gelation Mechanisms of Deoxycholate with Inorganic Additives: Hydrogen Bonding and Electrostatic Interactions. J. Phys. Chem. B.

[154] Li G., Hu Y., Sui J., Song A., Hao J. (2016). Hydrogelation and Crystallization of Sodium Deoxycholate Controlled by Organic Acids. Langmuir.

[155] McNeel K.E., Das S., Siraj N., Negulescu I.I., Warner I.M. (2015). Sodium Deoxycholate Hydrogels: Effects of Modifications on Gelation, Drug Release, and Nanotemplating. J. Phys. Chem. B.

[156] Song S., Feng L., Song A., Hao J. (2012). Room-Temperature Super Hydrogel as Dye Adsorption Agent. J. Phys. Chem. B.

[157] Sun X., Xin X., Tang N., Guo L., Wang L., Xu G. (2014). Manipulation of the Gel Behavior of Biological Surfactant Sodium Deoxycholate by Amino Acids. J. Phys. Chem. B.

[158] di Gregorio M.C., Shimon L.J.W., Brumfeld V., Houben L., Lahav M., van der Boom M.E. (2020). Emergence of Chirality and Structural Complexity in Single Crystals at the Molecular and Morphological Levels. Nat. Commun..

[159] Salzillo T., Giunchi A., Masino M., Bedoya-Martĺnez N., Della Valle R.G., Brillante A., Girlando A., Venuti E. (2018). An Alternative Strategy to Polymorph Recognition at Work: The Emblematic Case of Coronene. Cryst. Growth Des..

[160] Qiao Y., Lin Y., Wang Y., Yang Z., Liu J., Zhou J., Yan Y., Huang J. (2009). Metal-Driven Hierarchical Nanohelices. Nano Lett..

[161] Qiao Y., Wang Y., Yang Z., Lin Y., Huang J. (2011). Self-Templating of Metal-Driven Supramolecular Self-Assembly: A General Approach toward 1D Inorganic Nanotubes. Chem. Mater..

[162] Qiao Y., Lin Y., Zhang S., Huang J. (2011). Lanthanide-Containing Photoluminescent Materials: From Hybrid Hydrogel to Inorganic Nanotubes. Chem. A Eur. J..

[163] Laishram R., Bhowmik S., Maitra U. (2015). White Light Emitting Soft Materials from Off-the-Shelf Ingredients. J. Mater. Chem. C.

[164] Laishram R., Maitra U. (2018). Bile Salt-Derived Eu3+ Organogel and Hydrogel: Water-Enhanced Luminescence of Eu^3+^ in a Gel Matrix. ChemistrySelect.

[165] Wang H., Song S., Hao J., Song A. (2015). Hydrogels Triggered by Metal Ions as Precursors of Network CuS for DNA Detection. Chem. A Eur. J..

[166] Shen J., Wang Y., Xin X., Xu G., Li W., Wang L., Jia C. (2014). Studies on the Gel Behavior and Luminescence Properties of Biological Surfactant Sodium Deoxycholate/Rare-Earth Salts Mixed Systems. J. Colloid Interface Sci..

[167] Cheng C.Y., Oh H., Wang T.Y., Raghavan S.R., Tung S.H. (2014). Mixtures of Lecithin and Bile Salt Can Form Highly Viscous Wormlike Micellar Solutions in Water. Langmuir.

[168] Markina A.A., Ivanov V.A., Komarov P.V., Khokhlov A.R., Tung S.H. (2017). Self-Assembly of Lecithin and Bile Salt in the Presence of Inorganic Salt in Water: Mesoscale Computer Simulation. J. Phys. Chem. B.

[169] Cautela J., Giustini M., Pavel N.V., Palazzo G., Galantini L. (2017). Wormlike Reverse Micelles in Lecithin/Bile Salt/Water Mixtures in Oil. Colloids Surf. A Phys. Eng. Asp..

[170] Njauw C.W., Cheng C.Y., Ivanov V.A., Khokhlov A.R., Tung S.H. (2013). Molecular Interactions between Lecithin and Bile Salts/Acids in Oils and Their Effects on Reverse Micellization. Langmuir.

[171] Jean B., Oss-Ronen L., Terech P., Talmon Y. (2005). Monodisperse Bile-Salt Nanotubes in Water: Kinetics of Formation. Adv. Mater..

[172] Terech P., Velu S.K.P., Pernot P., Wiegart L. (2012). Salt Effects in the Formation of Self-Assembled Lithocholate Helical Ribbons and Tubes. J. Phys. Chem. B.

[173] Zhang X., Zou J., Tamhane K., Kobzeff F.F., Fang J. (2010). Self-Assembly of PH-Switchable Spiral Tubes: Supramolecular Chemical Springs. Small.

[174] Rhodes S., Liang W., Wang X., Reddy N.R., Fang J. (2020). Transition from H-Aggregate Nanotubes to J-Aggregate Nanoribbons. J. Phys. Chem. C.

[175] Zhang X., Bera T., Liang W., Fang J. (2011). Longitudinal Zipping/Unzipping of Self-Assembled Organic Tubes. J. Phys. Chem. B.

[176] D’Alagni M., Delfini M., Galantini L., Giglio E. (1992). A Study of the Interaction of Bilirubin Wtth Sodium Deoxycholate in Aqueous Solutions. J. Phys. Chem..

[177] D’Alagni M., D’Archivio A.A., Giglio E., Scaramuzza L. (1994). Structure of Sodium and Rubidium Taurodeoxycholate Micellar Aggregates and Their Interaction Complexes with Bilirubin-IXα. J. Phys. Chem..

[178] Meier A.R., Yehl J.B., Eckenroad K.W., Manley G.A., Strein T.G., Rovnyak D. (2018). Stepwise Aggregation of Cholate and Deoxycholate Dictates the Formation and Loss of Surface-Available Chirally Selective Binding Sites. Langmuir.

[179] Singh K., Chauhan S. (2020). Temperature Dependent Micellar Behaviour of Sodium Cholate and Sodium Deoxycholate in the Presence of Ceftriaxone Sodium: A Physicochemical Study. J. Mol. Liq..

[180] Zhang Q., Ma X., Ward A., Hong W.X., Jaakola V.P., Stevens R.C., Finn M.G., Chang G. (2007). Designing Facial Amphiphiles for the Stabilization of Integral Membrane Proteins. Angew. Chem. Int. Ed..

[181] Gubitosi M., Trillo J.V., Alfaro Vargas A., Pavel N.V., Gazzoli D., Sennato S., Jover A., Meijide F., Galantini L. (2014). Characterization of Carbon Nanotube Dispersions in Solutions of Bile Salts and Derivatives Containing Aromatic Substituents. J. Phys. Chem. B.

[182] di Gregorio M.C., Varenik M., Gubitosi M., Travaglini L., Pavel N.V., Jover A., Meijide F., Regev O., Galantini L. (2015). Multi Stimuli Response of a Single Surfactant Presenting a Rich Self-Assembly Behavior. RSC Adv..

[183] di Gregorio M.C., Pavel N.V., Jover A., Meijide F., Vázquez Tato J., Soto Tellini V.H., Vargas A.A., Regev O., Kasavi Y., Schillén K. (2013). PH Sensitive Tubules of a Bile Acid Derivative: A Tubule Opening by Release of Wall Leaves. Phys. Chem. Chem. Phys..

[184] Galantini L., Leggio C., Jover A., Meijide F., Pavel N.V., Soto Tellini V.H., Vázquez Tato J., Di Leonardo R., Ruocco G. (2009). Kinetics of Formation of Supramolecular Tubules of a Sodium Cholate Derivative. Soft Matter.

[185] Vázquez Tato J., Meijide F., Antelo A., Alvarez Alcalde M., Jover A., Galantini L., Pavel N.V. (2010). Supramolecular Structures Generated by a P-Tert-Butylphenylamide Derivative of Deoxycholic Acid. from Planar Sheets to Tubular Structures through Helical Ribbons. Langmuir.

[186] Meijide F., Trillo J.V., de Frutos S., Galantini L., Pavel N.V., Soto V.H., Jover A., Vázquez Tato J. (2012). Formation of Tubules by P-Tert-Butylphenylamide Derivatives of Chenodeoxycholic and Ursodeoxycholic Acids in Aqueous Solution. Steroids.

[187] Margulis-Goshen K., di Gregorio M.C., Pavel N.V., Abezgauz L., Danino D., Vázquez Tato J., Soto Tellini V.H., Magdassi S., Galantini L. (2013). Drug-Loaded Nanoparticles and Supramolecular Nanotubes Formed from a Volatile Microemulsion with Bile Salt Derivatives. Phys. Chem. Chem. Phys..

[188] Trillo J.V., Jover A., Galantini L., Tato J.V., Soto V.H., Meijide F., di Gregorio M.C., de Frutos S. (2013). Self-Aggregation Mechanism of a Naphthylamide Cationic Derivative of Cholic Acid. From Fibers to Tubules. RSC Adv..

[189] Travaglini L., D’Annibale A., Schillén K., Olsson U., Sennato S., Pavel N.V., Galantini L. (2012). Amino Acid–Bile Acid Based Molecules: Extremely Narrow Surfactant Nanotubes Formed by a Phenylalanine-Substituted Cholic Acid. Chem. Commun..

[190] Travaglini L., D’Annibale A., di Gregorio M.C., Schillén K., Olsson U., Sennato S., Pavel N.V., Galantini L. (2013). Between Peptides and Bile Acids: Self-Assembly of Phenylalanine Substituted Cholic Acids. J. Phys. Chem. B.

[191] Travaglini L., Gubitosi M., di Gregorio M.C., Pavel N.V., D’Annibale A., Giustini M., Soto Tellini V.H., Vázquez Tato J., Obiols-Rabasa M., Bayati S. (2014). On the Self-Assembly of a Tryptophan Labeled Deoxycholic Acid. Phys. Chem. Chem. Phys..

[192] Travaglini L., Gubitosi M., di Gregorio M.C., D’Annibale A., Meijide F., Giustini M., Sennato S., Obiols-Rabasa M., Schillén K., Pavel N.V. (2015). A Tryptophan-Substituted Cholic Acid: Expanding the Family of Labelled Biomolecules. Colloids Surf. A Phys. Eng. Asp..

[193] Gubitosi M., Travaglini L., D’Annibale A., Pavel N.V., Vázquez Tato J., Obiols-Rabasa M., Sennato S., Olsson U., Schillén K., Galantini L. (2014). Sugar-Bile Acid-Based Bolaamphiphiles: From Scrolls to Monodisperse Single-Walled Tubules. Langmuir.

[194] Gubitosi M., D’Annibale A., Schillén K., Olsson U., Pavel N.V., Galantini L. (2017). On the Stability of Lithocholate Derivative Supramolecular Tubules. RSC Adv..

[195] Soto Tellini V.H., Jover A., Meijide F., Vázquez Tato J., Galantini L., Pavel N.V. (2007). Supramolecular Structures Generated by a P-Tert-Butylphenylamide Derivative of Cholic Acid: From Vesicles to Molecular Tubes. Adv. Mater..

[196] Manghisi N., Leggio C., Jover A., Meijide F., Pavel N.V., Tellini V.H.S., Tato J.V., Agostino R.G., Galantini L. (2010). Catanionic Tubules with Tunable Charge. Angew. Chem. Int. Ed..

[197] di Gregorio M.C., Severoni E., Travaglini L., Gubitosi M., Sennato S., Mura F., Redondo-Gómez C., Jover A., Pavel N.V., Galantini L. (2018). Bile Acid Derivative-Based Catanionic Mixtures: Versatile Tools for Superficial Charge Modulation of Supramolecular Lamellae and Nanotubes. Phys. Chem. Chem. Phys..

[198] Gubitosi M., Travaglini L., di Gregorio M.C., Pavel N.V., Vazquez-Tato J., Sennato S., Olsson U., Schillén K., Galantini L. (2015). Tailoring Supramolecular Nanotubes by Bile Salt Based Surfactant Mixtures. Angew. Chem. Int. Ed..

[199] Cautela J., Lattanzi V., Månsson L., Galantini L., Crassous J.J. (2018). Sphere–Tubule Superstructures through Supramolecular and Supracolloidal Assembly Pathways. Small.

[200] Gubitosi M., Meijide F., D’Annibale A., Vázquez Tato J., Jover A., Galantini L., Travaglini L., di Gregorio M.C., Pavel N.V. (2016). Crystal Structure of a Lithium Salt of a Glucosyl Derivative of Lithocholic Acid. Steroids.

[201] Cautela J., Severoni E., Redondo-Gómez C., di Gregorio M.C., Del Giudice A., Sennato S., Angelini R., D’Abramo M., Schillén K., Galantini L. (2020). Substituent Position and Orientation on the Self-Assembly of Steroid Surfactant Isomers. Colloids Surf. B..

[202] di Gregorio M.C., Ranjan P., Houben L., Shimon L.J.W., Rechav K., Lahav M., Van Der Boom M.E. (2018). Metal-Coordination-Induced Fusion Creates Hollow Crystalline Molecular Superstructures. J. Am. Chem. Soc..

[203] Travaglini L., di Gregorio M.C., Severoni E., Annibale A.D., Sennato S., Tardani F., Giustini M., Gubitosi M., Del A., Galantini L. (2019). Deoxycholic Acid and L -Phenylalanine Enrich Their Hydrogel Properties When Combined in a Zwitterionic Derivative. J. Colloid Interface Sci..

[204] Pore V.S., Agalave S.G., Pharande S.G., Patil P.A., Kotmale A.S. (2015). Bile Acid Hydrazides: Gelation, Structural, Physical and Spectroscopic Properties. New J. Chem..

[205] Travaglini L., Giordano C., D’Annibale A., Gubitosi M., di Gregorio M.C., Schillén K., Stefanucci A., Mollica A., Pavel N.V., Galantini L. (2017). Twisted Nanoribbons from a RGD-Bearing Cholic Acid Derivative. Colloids Surf. B..

[206] Noponen V., Valkonen A., Lahtinen M., Salo H., Sievänen E. (2013). Self-Assembly Properties of Bile Acid Derivatives of L-Cysteine, L-Valine and L-Serine Alkyl Esters. Supramol. Chem..

[207] Sajisha V.S., Maitra U. (2014). Remarkable Isomer-Selective Gelation of Aromatic Solvents by a Polymorph of a Urea-Linked Bile Acid– Amino Acid Conjugate. RSC Adv..

[208] Maity M., Maitra U. (2017). Supramolecular Gels from Conjugates of Bile Acids and Amino Acids and Their Applications. Eur. J. Org. Chem..

[209] di Gregorio M.C., Pavel N.V., Miragaya J., Jover A., Meijide F., Vázquez Tato J., Soto Tellini V.H., Galantini L. (2013). Catanionic Gels Based on Cholic Acid Derivatives. Langmuir.

[210] Chiang J.Y.L. (2013). Bile Acid Metabolism and Signaling. Compr. Physiol..

[211] De Aguiar Vallim T.Q., Tarling E.J., Edwards P.A. (2013). Pleiotropic Roles of Bile Acids in Metabolism. Cell Metab..

[212] Wahlström A., Sayin S.I., Marschall H.U., Bäckhed F. (2016). Intestinal Crosstalk between Bile Acids and Microbiota and Its Impact on Host Metabolism. Cell Metab..

[213] Wu Y., Zhou A., Tang L., Lei Y., Tang B., Zhang L. (2020). Bile Acids: Key Regulators and Novel Treatment Targets for Type 2 Diabetes. J. Diabetes Res..

[214] Grant S.M., Demorrow S. (2020). Bile Acid Signaling in Neurodegenerative and Neurological Disorders. Int. J. Mol. Sci..

